# Perovskite quantum dot-based gas sensors for environmental monitoring: mechanisms, materials, and perspectives on next-generation pollution control

**DOI:** 10.1039/d5ra07219k

**Published:** 2025-12-02

**Authors:** Suleiman Ibrahim Mohammad, A. K. Kareem, Zyad Shaaban, M. Sudhakara Reddy, Asokan Vasudevan, Fadhil Faez Sead, D. S. Jayalakshmi, Sanjeev Kumar, Satish Choudhury, Ahmad Mohebi

**Affiliations:** a Electronic Marketing and Social Media, Economic and Administrative Sciences Zarqa University Zarqa Jordan; b Research Follower, INTI International University 71800 Negeri Sembilan Putra Nilai Malaysia; c Biomedical Engineering Department, College of Engineering, Al-Mustaqbal University Hillah 51001 Babil Iraq; d Department of Computer Science, University College of Duba, University of Tabuk Duba 71911 Saudi Arabia; e Department of Physics & Electronics, School of Sciences, JAIN (Deemed to Be University) Bangalore Karnataka India; f Faculty of Business and Communications, INTI International University 71800 Negeri Sembilan Malaysia; g Shinawatra University 99 Moo 10, Bangtoey, Samkhok, Pathum Thani 12160 Thailand; h Department of Dentistry, College of Dentistry, The Islamic University Najaf Iraq; i Department of Medical Analysis, Medical Laboratory Technique College, The Islamic University of Al Diwaniyah Al Diwaniyah Iraq; j Department of Medical Analysis, Medical Laboratory Technique College, The Islamic University of Babylon Babylon Iraq; k Department of Physics, Sathyabama Institute of Science and Technology Chennai Tamil Nadu India; l Department of Physics, University Institute of Sciences, Chandigarh University Mohali Punjab India; m Department of Electrical & Electronics Engineering, Siksha ‘O' Anusandhan (Deemed to Be University) Bhubaneswar Odisha-751030 India; n Department of chemistry, Young Researchers and Elite Club, Tehran Branch, Islamic Azad University Tehran Iran a.mohebiacademic@gmail.com; o Sharda School of Engineering and Science, Sharda University Greater Noida India

## Abstract

Perovskite quantum dots (PQDs) have recently emerged as transformative nanomaterials for gas sensing, offering exceptional optoelectronic properties, high surface-to-volume ratios, and compositional tunability. This is the first comprehensive review that systematically analyzes gas sensing technologies based on PQDs, with a particular emphasis on their relevance to environmental monitoring and pollution control. We summarize the latest advances in sensing mechanisms—including fluorescence quenching and enhancement, ratiometric detection, and chemiresistive/conductometric responses—and evaluate how synthesis strategies, surface ligand engineering, and hybrid architectures govern sensor performance. Key applications are critically assessed in the detection of toxic gases (NO_2_, NH_3_, H_2_S, SO_2_), volatile organic compounds, oxygen, and humidity, all of which are central to air quality assessment and environmental safety. Special focus is given to stability challenges under ambient and humid conditions, the environmental toxicity of lead-based PQDs, and mitigation strategies such as encapsulation, ligand engineering, and the development of lead-free alternatives. By integrating nanoscale material design with real-world environmental needs, this review not only consolidates current knowledge but also provides forward-looking perspectives for developing robust, selective, and sustainable PQD-based sensors for next-generation environmental monitoring systems.

## Abstract 2

This is the first comprehensive review that systematically analyzes gas sensing technologies based on PQDs, with a particular emphasis on their relevance to environmental monitoring and pollution control.

## Graphical abstract

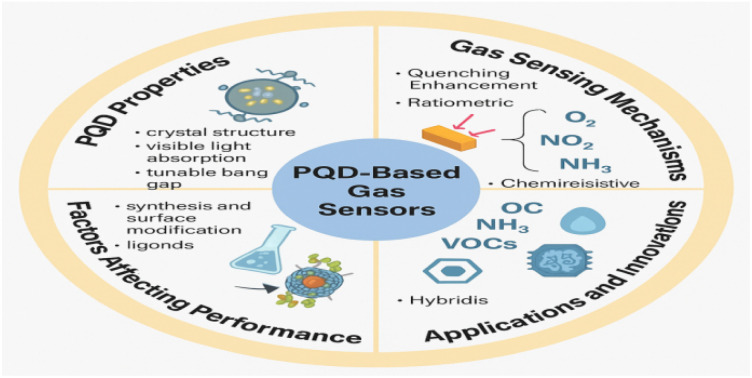

## 1. Introduction

Perovskite quantum dots (PQDs) have emerged as a transformative class of nanomaterials with exceptional promise in optoelectronic and sensing applications.^1–3^ Structurally defined by the formula ABX_3_, where A is a monovalent cation (*e.g.*, Cs^+^, MA^+^, FA^+^), B is a divalent metal cation (commonly Pb^2+^ or Sn^2+^), and X is a halide anion (Cl^−^, Br^−^, or I^−^), these materials exhibit highly tunable optical and electronic properties.^4–6^ Quantum confinement effects, resulting from their ultrasmall size (typically below 10 nm), lead to discrete energy levels and strong photoluminescence, which can be finely adjusted by modifying their composition.^7,8^ High photoluminescence quantum yields (PLQYs), narrow emission linewidths, and low defect densities have enabled PQDs to play a leading role in fields such as photovoltaics, light-emitting diodes, and most recently, chemical and gas sensing.^9,10^ Gas sensing is a critical component in various sectors, including environmental monitoring, industrial process control, occupational safety, and biomedical diagnostics. The increasing demand for sensitive, selective, low-power, and miniaturized sensors has driven research toward novel materials with advanced functionality.^11–13^ Traditional gas sensors—such as those based on metal oxides^14,15^ or carbon nanomaterials^16,17^—often require high operating temperatures and suffer from limited selectivity and slow response–recovery dynamics.

PQDs offer an attractive alternative due to their unique optoelectronic behavior under ambient conditions, enabling rapid, low-temperature detection of diverse analytes including toxic gases (*e.g.*, NO_2_, NH_3_, H_2_S, SO_2_), volatile organic compounds (*e.g.*, ethanol, methanol, formaldehyde), oxygen, and humidity. Their high surface-to-volume ratio facilitates gas adsorption, while their ionic crystal structure allows for dynamic interactions with gas molecules, making them especially effective in transducing chemical changes into optical or electrical signals.^18–20^ Despite their potential, PQD-based gas sensors face several critical challenges that must be addressed to enable real-world deployment. A primary limitation lies in their environmental instability. The ionic nature of perovskites makes them vulnerable to degradation under moisture, heat, and light, leading to phase transitions, reduced PLQY, and poor long-term durability.^20,21^ In particular, exposure to humidity and reactive gases can cause irreversible structural damage. Moreover, the widespread use of lead-based PQDs, such as CsPbBr_3_ or MAPbI_3_, raises environmental and health concerns due to potential toxicity. These issues necessitate the development of stabilization strategies—such as surface passivation, ligand engineering, and encapsulation in porous or polymeric matrices—as well as the exploration of lead-free alternatives like CsSnX_3_ and Cs_2_AgBiX_6_.^22,23^

**Fig. 1:**
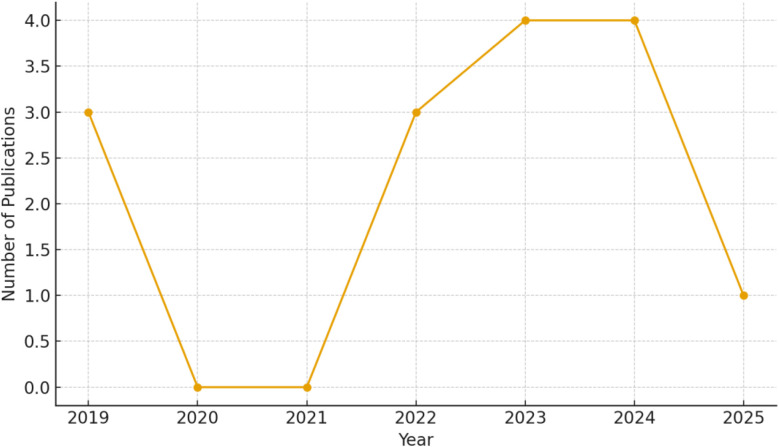
The annual publication trend of PQD-based gas sensing research between 2019 and 2025.

Although PQDs have been widely explored for optoelectronic applications, their utilization in gas sensing remains comparatively limited. To better illustrate the research progress in this emerging field, a bibliometric analysis of all available PQD-based gas sensing studies from 2019 to 2025 was conducted (Fig. 1). The collected data reveal a clear growth trend over recent years, indicating increasing scientific interest in developing PQD-based sensing technologies.

**Fig. 2:**
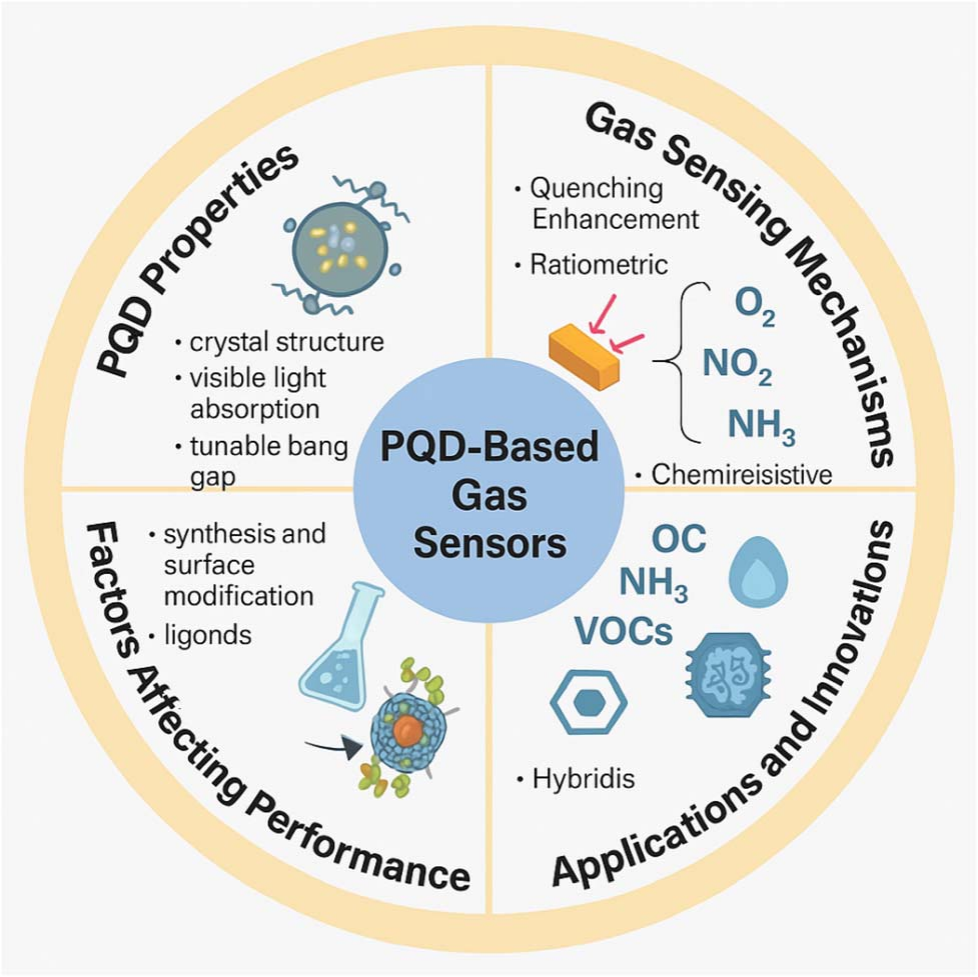
Overview of PQD-based gas sensors, highlighting key properties, sensing mechanisms, performance factors, and applications in gas detection.

This review provides a comprehensive and systematic analysis of gas sensing technologies based on PQDs. It begins by outlining the fundamental structural, compositional, and optoelectronic features of PQDs that render them suitable for gas sensing applications. Various sensing mechanisms are then discussed, including fluorescence quenching and enhancement, ratiometric approaches, and chemiresistive and conductometric detection. The influence of synthesis techniques, ligand chemistry, and hybrid material design on sensor performance—particularly in terms of selectivity, sensitivity, response time, and stability—is examined in detail. Applications in detecting inorganic gases, VOCs, humidity, and oxygen are critically reviewed, supported by performance metrics such as LOD, response/recovery times, and operational robustness. Furthermore, the review highlights recent design innovations, including hybridization with metal oxides, ratiometric sensing platforms, and machine learning-based signal processing (Fig. 2). By synthesizing the latest advances in materials engineering, sensor design, and application development, this work aims to serve as a foundational reference for scientists and engineers seeking to harness the potential of PQDs in next-generation gas sensing platforms. The review not only consolidates current knowledge but also identifies critical knowledge gaps and outlines pathways for future innovation. Ultimately, PQD-based gas sensors represent a promising frontier for achieving sensitive, selective, stable, and scalable detection systems across environmental, biomedical, and industrial domains.

## 2. Fundamentals of PQDs for sensing

PQDs have emerged as a transformative class of nanomaterials in the realm of gas sensing, owing to their exceptional optoelectronic properties, structural versatility, and tunable surface chemistry. This section elucidates the foundational aspects of PQDs that underpin their efficacy in gas sensing applications, including their structural and compositional diversity, optoelectronic properties tailored for sensing, synthesis techniques that influence their performance, and intrinsic stability challenges along with initial mitigation strategies. These fundamentals provide the necessary context for understanding the mechanisms and applications of PQD-based gas sensors.

### 2.1. Structural and compositional diversity of PQDs

**Fig. 3:**
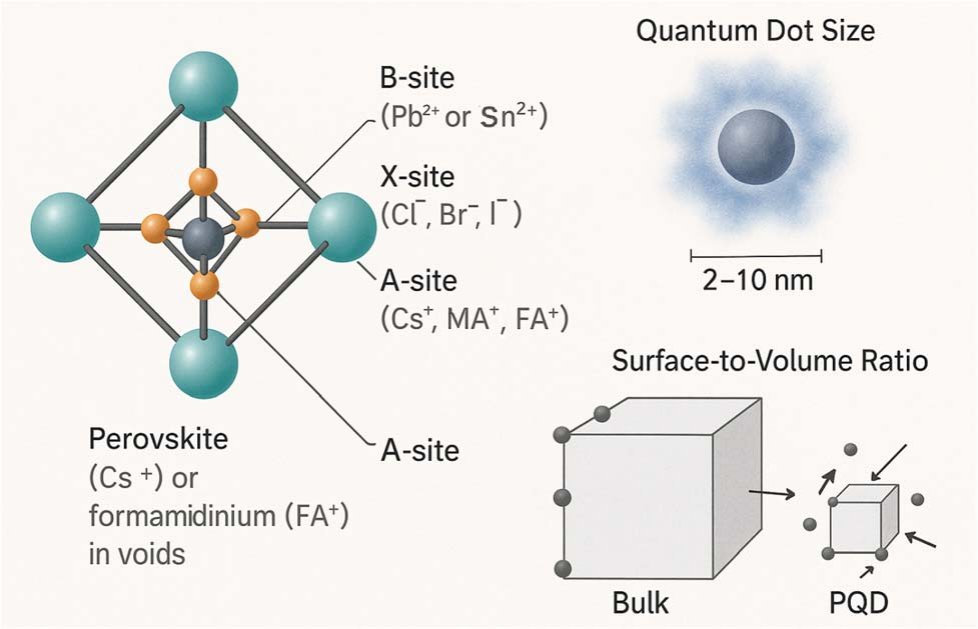
PQDs adopt the ABX_3_ formula with A = Cs^+^, MA^+^, or FA^+^; B=Pb^2+^ or Sn^2+^; and X = Cl^−^, Br^−^, or I^−^. The structure features corner-sharing BX_6_ octahedra and A-site cations in the voids. Nanoscale size (2–10 nm) enables quantum confinement and boosts gas sensing.

PQDs adopt the general formula ABX_3_, where A is a monovalent cation (*e.g.*, cesium, Cs^+^; methylammonium, MA^+^; formamidinium, FA^+^), B is a divalent metal cation (*e.g.*, Pb^2+^, Sn^2+^), and X is a halide anion (*e.g.*, Cl^−^, Br^−^, I^−^). The perovskite structure consists of a cubic or pseudo-cubic lattice, where the B cation is coordinated octahedrally with six X anions, forming BX_6_ octahedra, and the A cation occupies the cuboctahedral voids.^24,25^ This structural framework imparts PQDs with remarkable flexibility, allowing compositional tuning to modulate their electronic and optical properties for gas sensing. The nanoscale dimensions of PQDs, typically ranging from 2 to 10 nm, induce pronounced quantum confinement effects, thereby enhancing their functionality in gas sensing applications. This reduced dimensionality significantly increases the surface-to-volume ratio, resulting in a higher density of accessible active sites for gas adsorption and interaction. Furthermore, compositional engineering—such as halide mixing (*e.g.*, CsPb(Br/I)_3_) and A-site cation substitution (*e.g.*, methylammonium [MA^+^] with formamidinium [FA^+^])—enables precise modulation of the bandgap and emission properties^26,27^ (Fig. 3).

These tunable optoelectronic characteristics are particularly advantageous for fluorescence-based sensing, where specific emission wavelengths correspond to tailored analyte responses. For example, CsPbBr_3_ PQDs typically exhibit green photoluminescence (PL ∼ 520 nm), whereas CsPbI_3_ PQDs emit in the red region (∼700 nm), facilitating sensor designs optimized for selective optical detection of target gas species. Furthermore, the ionic nature of the perovskite lattice facilitates dynamic surface interactions with gas molecules, such as ammonia (NH_3_) or nitrogen dioxide (NO_2_), which can modulate luminescence or conductivity. However, this ionic character also renders PQDs sensitive to environmental factors, necessitating structural modifications to enhance stability.^28,29^ The versatility of PQDs extends to lead-free compositions (*e.g.*, CsSnX_3_, Cs_2_AgBiX_6_), which address toxicity concerns while maintaining sensing capabilities, albeit with challenges in achieving comparable PL efficiency.

### 2.2. Optoelectronic properties enabling sensing applications

The optoelectronic properties of PQDs are central to their effectiveness in gas sensing, particularly in fluorescence-based and chemiresistive sensing modalities. PQDs exhibit high PLQY, often exceeding 90% for CsPbBr_3_, due to their direct bandgap and low defect densities. This high PLQY enables sensitive detection of gas molecules that induce quenching or enhancement of fluorescence through charge transfer or surface adsorption.^30,31^ For example, electron-donating gases like NH_3_ can passivate surface defects, enhancing PL intensity, while electron-withdrawing gases like NO_2_ can quench emission by extracting charge carriers.^32–34^ The tunable bandgap of PQDs, ranging from 1.8 eV (CsPbI_3_) to 3.0 eV (CsPbCl_3_), allows for wavelength-specific responses to gas analytes, facilitating selective detection in fluorescence-based sensors. The narrow emission linewidths (full width at half maximum, FWHM, of 12–40 nm) ensure high spectral resolution, enabling ratiometric sensing approaches where changes in emission intensity or peak position signal gas presence. Additionally, the high exciton binding energy in PQDs (up to 100 meV) enhances radiative recombination, making them ideal for detecting subtle changes in surface chemistry induced by gas interactions.^24,33^

**Fig. 4:**
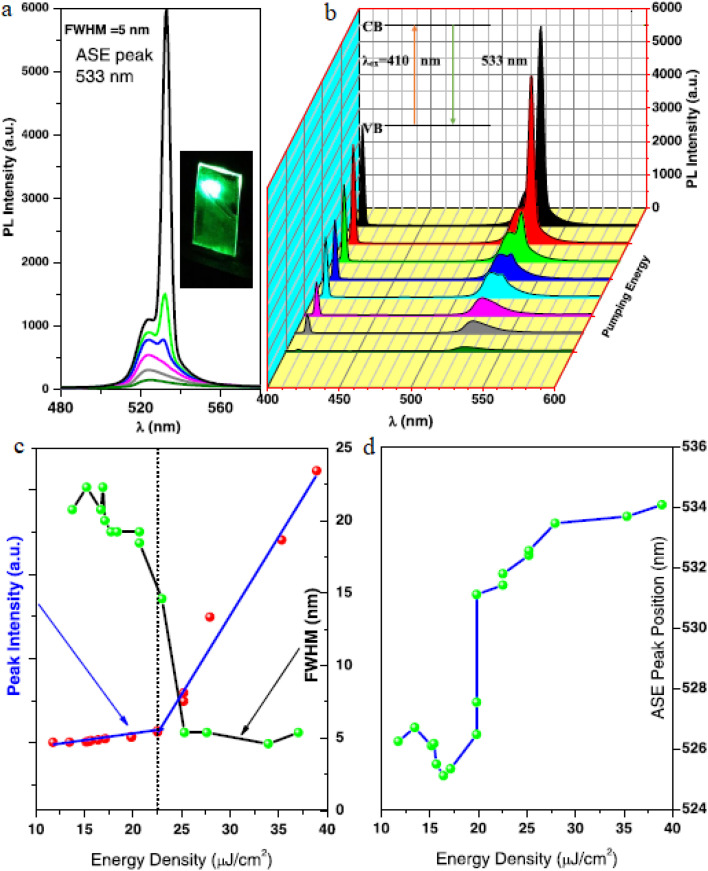
(a) Evolution of PL spectra with varying pulse energy density and (b) 3D diagram of pump fluence *versus* PL intensity for a CsPbBr_3_ PQD thin film under 410 nm excitation. (c) Integrated PL intensity and FWHM behavior and (d) ASE peak position as functions of pulse energy density for the CsPbBr_3_ PQD thin film under 410 nm excitation. Reprinted with permission from ref. 6. Copyright 2020 American Chemical Society.

Fig. 4a displays the PL spectra evolution of a CsPbBr_3_ PQD thin film under 410 nm excitation as pulse energy density varies. A distinct amplified spontaneous emission (ASE) peak at 533 nm with a FWHM of 5 nm is evident, as highlighted in the inset image, reflecting high radiative efficiency and low defect density. This aligns with the material's high PLQY, enhancing its sensitivity to gas-induced fluorescence changes, a key attribute for optoelectronic sensing applications. Fig. 4b presents a 3D diagram illustrating the pump fluence *versus* PL intensity relationship for the CsPbBr_3_ PQD thin film. The nonlinear increase in PL intensity with energy density indicates a threshold for ASE onset, consistent with the tunable bandgap properties that enable wavelength-specific responses. This visualization supports the material's potential for ratiometric sensing, where shifts in emission characteristics can signal gas presence, reinforcing its utility in selective detection. Fig. 4c and d detail the optoelectronic response with respect to pulse energy density. In 4c, the integrated PL intensity and FWHM show a sharp rise and narrowing beyond a threshold, indicating amplified emission and reduced non-radiative losses, supported by the high exciton binding energy. In 4d, the ASE peak position shifts from 536 nm to 526 nm with increasing energy density, reflecting electronic structure changes that enhance detection of subtle surface chemistry alterations induced by gas interactions.

In chemiresistive sensing, PQDs demonstrate remarkable electrical properties, including high carrier mobility (up to 4500 cm^2^ V^−1^ s^−1^ in CsPbBr_3_ thin films) and tunable conductivity. Gas molecules adsorbed on PQD surfaces can alter charge carrier concentrations, modulating resistance. For instance, oxidizing gases like NO_2_ increase resistance by trapping electrons, while reducing gases like H_2_S decrease resistance by donating electrons. The large surface area of PQDs amplifies these effects, enabling low LODs in the ppb range for gases like hydrogen sulfide (H_2_S).^35–37^ The defect tolerance of PQDs, arising from their electronic structure where defect states lie within or near the conduction and valence bands, minimizes non-radiative recombination, enhancing both optical and electrical sensitivity. However, surface defects, such as halide vacancies, can act as active sites for gas adsorption, necessitating careful control during synthesis to balance sensitivity and stability. Table 1 shows the structural, compositional, and optoelectronic properties of PQDs for gas sensing applications.

**Table 1:** Structural, compositional, and optoelectronic properties of PQDs for gas sensing applications

Property	Description	Impact on gas sensing	Specific features	Advantages	Limitations	Related gas sensing applications	Ref.
Structural diversity	ABX_3_ perovskite lattice (A: Cs^+^, MA^+^, FA^+^; B: Pb^2+^, Sn^2+^; X: Cl^−^, Br^−^, I^−^); cubic/pseudo-cubic with BX_6_ octahedra	Enables compositional tuning for tailored optical/electrical responses to gases	Size: 2–10 nm; high surface-to-volume ratio; mixed-halide (*e.g.*, CsPb(Br/I)_3_)	Large active sites for gas interactions; tunable emission (520 nm for CsPbBr_3_, 700 nm for CsPbI_3_)	Ionic lattice sensitive to moisture, requiring stabilization	NH_3_ (fluorescence enhancement), NO_2_ (quenching), H_2_S (LOD 250 ppb)	24 and 25
Compositional versatility	Flexibility in A, B, X components; lead-free options (*e.g.*, CsSnX_3_, Cs_2_AgBiX_6_)	Allows bandgap tuning and reduced toxicity for specific gas detection	Bandgap: 1.8 eV (CsPbI_3_) to 3.0 eV (CsPbCl_3_); lead-free reduces environmental impact	Tailored sensors for fluorescence-based detection; eco-friendly alternatives	Lower PLQY in lead-free PQDs; stability challenges	NH_3_ (LOD 0.5 ppm), SO_2_ (LOD 1 ppm)	26 and 27
Photoluminescence	High PLQY (up to 90% for CsPbBr_3_); narrow FWHM (12–40 nm)	Enables sensitive fluorescence-based sensing *via* quenching or enhancement	Direct bandgap; low defect density; high exciton binding energy (∼100 meV)	High sensitivity to gas-induced PL changes; spectral resolution for ratiometric sensing	Susceptible to photo-induced degradation; requires passivation	NH_3_ (PL enhancement), NO_2_ (quenching, response 53 at 5 ppm), O_2_ (sensitivity 12.7)	28 and 29
Electrical conductivity	High carrier mobility (up to 4500 cm^2^ V^−1^ s^−1^ in CsPbBr_3_); tunable conductivity	Facilitates chemiresistive sensing through gas-induced resistance changes	Surface defects as active sites; defect-tolerant electronic structure	Low LODs (*e.g.*, H_2_S at 250 ppb); amplified response due to large surface area	Surface defects may reduce stability; needs balanced synthesis	H_2_S (response 0.58), TEA (response 52.92 at 60 °C), NO_2_ (increased resistance)	30 and 31
Quantum confinement	Nanoscale size (2–10 nm) induces quantum confinement, enhancing optical properties	Increases sensitivity to gas-induced optical changes *via* modified bandgap	Tunable emission wavelengths; enhanced PL due to confinement effects	Precise control over optical responses for specific gases	Small size increases surface sensitivity to environmental degradation	NH_3_ (fluorescence turn-on), SO_2_ (LOD 1 ppm)	32 and 33
Defect tolerance	Defect states lie within/near conduction and valence bands, minimizing non-radiative recombination	Enhances both optical and electrical sensitivity for gas detection	Low non-radiative losses; halide vacancies as active sites for gas adsorption	High sensitivity in fluorescence and chemiresistive sensors	Excessive defects can reduce long-term stability	H_2_S (LOD 250 ppb), NO (sensitivity 6 at 1000 ppm)	24 and 29
Surface chemistry	Ionic lattice enables dynamic interactions with gas molecules (*e.g.*, NH_3_, NO_2_)	Modulates PL or conductivity for selective gas detection	Surface defects and ligand interactions tailor gas adsorption	High selectivity for electron-donating (NH_3_) or withdrawing (NO_2_) gases	Susceptible to analyte-induced degradation; requires surface passivation	NH_3_ (LOD 0.5 ppm), NO_2_ (response 53 at 5 ppm)	25 and 35

### 2.3. Synthesis techniques and their influence on sensing performance

The synthesis method of PQDs plays a central role in defining their suitability for gas sensing, as it directly impacts their size, crystallinity, surface chemistry, and stability. These parameters influence key sensing metrics such as PLQY, surface defect density, and gas adsorption behavior. Among the widely adopted methods are hot-injection, ligand-assisted reprecipitation (LARP), room-temperature co-precipitation, and solvothermal synthesis. Each offers distinct advantages depending on whether fluorescence- or resistance-based sensing is targeted.

#### 2.3.1. Hot-injection synthesis

Hot-injection is widely recognized for producing high-quality PQDs with narrow size distributions and superior optical properties. This method involves injecting cesium oleate into a hot (140–200 °C) solution containing lead halide and organic ligands (*e.g.*, oleic acid, oleylamine) under inert atmosphere. Rapid nucleation leads to uniform PQDs, typically 2–8 nm in size, with polydispersity below 5%. High crystallinity and PLQY (up to 90% for CsPbBr_3_) make these PQDs ideal for fluorescence-based gas sensing. This method minimizes surface defects, enhancing sensitivity in applications where PL changes signal gas presence, such as NH_3_ or NO_2_ detection. However, long-chain ligands used in the process can limit gas diffusion to active sites. Ligand exchange with shorter or zwitterionic molecules (*e.g.*, DDAB) can improve surface accessibility while maintaining colloidal stability.^28,38,39^ Despite high sensor performance, the need for strict temperature control and inert conditions hinders scalability.

#### 2.3.2. Ligand-assisted reprecipitation

**Fig. 5:**
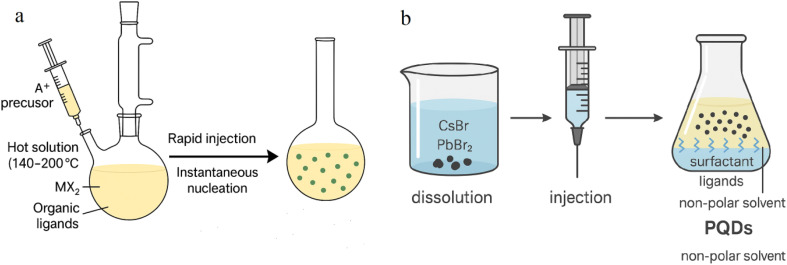
Methods for synthesizing PQDs: (a) hot-injection technique and (b) ligand-assisted reprecipitation approach.

LARP is a low-temperature, solution-based method suited for scalable PQD production. It involves dissolving Cs and Pb precursors in a polar solvent like DMF, then injecting the solution into a non-polar solvent (*e.g.*, toluene) containing surfactant ligands. This polarity shock triggers rapid nucleation. PQDs typically range from 2–10 nm, with PLQY between 70–85%. LARP-generated PQDs tend to exhibit higher surface defect densities than those from hot-injection, which can be beneficial for chemiresistive sensing by providing more active sites for gas adsorption. For instance, LARP-derived CsPbBr_3_ sensors achieved an H_2_S detection limit of 250 ppb. However, these same defects reduce stability under humid conditions, leading to PL quenching over time. Encapsulation strategies—such as embedding in silica aerogels—can mitigate degradation.^40–42^ The method's simplicity and tunability make it attractive for applications where rapid, cost-effective synthesis is required, though broader size distributions may limit fluorescence resolution (Fig. 5).

#### 2.3.3. Room-temperature Co-precipitation

Co-precipitation offers a simple and scalable route to PQD synthesis. Precursors are mixed in a single solvent (*e.g.*, DMF), followed by the gradual addition of a poor solvent (*e.g.*, acetone), which reduces solubility and induces nucleation. The process proceeds at ambient temperature, eliminating the need for high heat or inert atmospheres. Resulting PQDs are typically 4–12 nm with broader size distributions (10–15% polydispersity) and moderate PLQY (50–70%). While not ideal for high-performance fluorescence sensors, these PQDs are well-suited for resistance-based sensing. For example, co-precipitated CsPbBr_3_–In_2_O_3_ composites showed strong response (52.92) to triethylamine (TEA) at 60 °C. Stability can be further improved through microwave-assisted co-precipitation, which enhances crystallinity and reduces defects.^24,43,44^ However, short-chain ligands required for gas access can compromise long-term colloidal integrity, highlighting the need for optimized ligand selection.

#### 2.3.4. Solvothermal synthesis

**Fig. 6:**
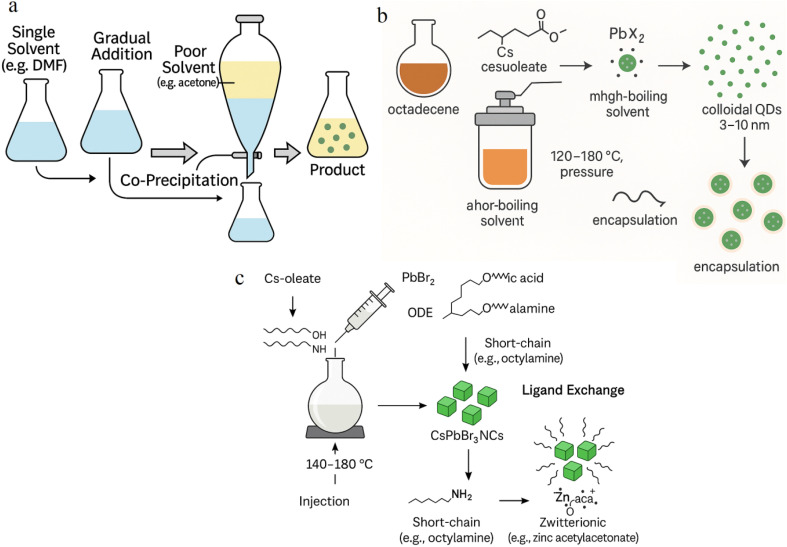
Alternative PQD synthesis strategies: (a) room-temperature Co-precipitation method, (b) solvothermal process, and (c) influence of ligands and post-synthesis adjustments.

Solvothermal synthesis is a promising approach for producing structurally stable PQDs, especially for use in humid or high-temperature environments. Precursors are dissolved in a high-boiling solvent (*e.g.*, octadecene) and heated in a sealed autoclave (120–180 °C) under pressure. The controlled environment promotes low-defect crystal growth, yielding PQDs with sizes of 3–10 nm and polydispersity of 3–7%. These PQDs demonstrate good moisture resistance and PLQY around 85%, suitable for both optical and resistive sensing. For example, solvothermally synthesized CsPbBr_3_ showed excellent humidity sensing with an LOD of 0.1% RH, and NO_2_ sensing with a chemiresistive response of 53 at 5 ppm. However, the method requires longer synthesis times (1–4 hours) and specialized high-pressure equipment, which increases cost and limits throughput. Proper ligand engineering remains critical to balance gas diffusion and environmental stability^26,30,45^ (Fig. 6).

#### 2.3.5. Ligand effects and post-synthesis modifications

Ligand selection during and after synthesis critically affects PQD sensor performance. Long-chain ligands such as oleic acid offer colloidal stability but inhibit gas interaction, reducing sensitivity in chemiresistive formats. Replacing them with short-chain (*e.g.*, octylamine) or zwitterionic ligands enhances gas access but may compromise environmental resistance. For instance, ethanol sensors using CsPbBr_3_ capped with zinc acetylacetonate achieved a 3 ppm detection limit under humid conditions due to improved surface interactions. Post-synthesis ligand exchange and encapsulation in matrices such as zeolites, polymers, or silica aerogels help protect PQDs from degradation while preserving surface reactivity. CsPbBr_3_ embedded in Fe-doped zeolite X retained 98% PL intensity after 100 days in humid air, enabling stable NH_3_ sensing. Hybridization with metal oxides (*e.g.*, ZnO, In_2_O_3_) further improves performance by promoting charge transfer and enhancing durability.^25,27,46^

### 2.4. Intrinsic stability issues and initial mitigation strategies

The primary limitation of PQDs in gas sensing is their intrinsic instability, particularly under exposure to moisture, heat, and light, which can degrade their optoelectronic properties. The ionic nature of the perovskite lattice makes PQDs susceptible to dissociation in humid environments, as water molecules coordinate with surface ions, leading to structural collapse and loss of luminescence.^26^ For gas sensing, this is particularly problematic, as many target gases (*e.g.*, NH_3_, H_2_O vapor) are detected in humid conditions, necessitating robust stabilization strategies. Thermal instability arises from the low formation energy of PQDs, causing phase transitions or decomposition above 100 °C.^47^ This limits their use in high-temperature industrial gas sensing applications unless mitigated. Photo-induced degradation, driven by prolonged exposure to UV or visible light, can generate halide vacancies, reducing PLQY and altering sensing responses.^28^ These challenges are exacerbated in gas sensing, where PQDs are exposed to reactive analytes that may accelerate degradation.^24^ Initial mitigation strategies focus on enhancing stability without compromising sensing performance. Surface passivation with short-chain or zwitterionic ligands (*e.g.*, didodecyldimethylammonium bromide) strengthens the PQD surface against moisture and gas-induced degradation while maintaining accessibility for analyte interactions.^48^

**Fig. 7:**
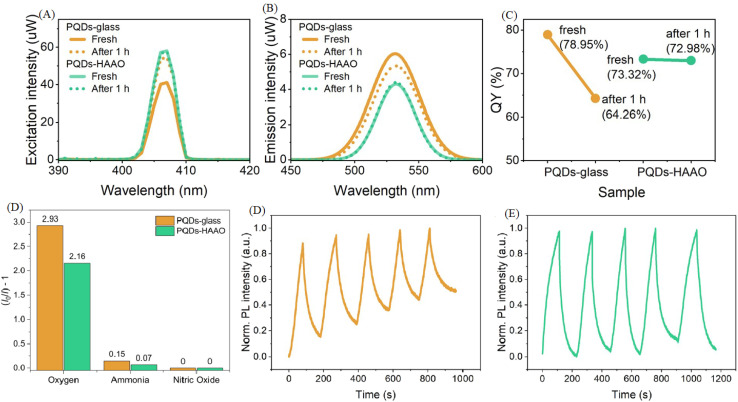
QY and gas-sensing performance of PQDs: (A) excitation spectra, (B) emission spectra, (C) QY after 1-hour oxygen exposure (∼20%), (D) response to 100% O_2_, 1000 ppm NH_3_, and NO, (E) PQDs-glass response–recovery cycles (100% O_2_/N_2_), (F) PQDs-HAAO response–recovery cycles (100% O_2_/N_2_). Reproduced with permission from ref. 48. Copyright 2025 Royal Society of Chemistry.

Fig. 7 provides a comprehensive analysis of the QY characteristics and gas-sensing performance of PQDs on different substrates, addressing their optoelectronic stability under various conditions.^48^ In panel A, the excitation spectra of PQDs-glass and PQDs-HAAO samples show a peak around 410 nm, indicating consistent absorption properties despite substrate differences. Panel B presents the emission spectra, revealing a broad emission band centered near 520 nm for both samples, with PQDs-HAAO exhibiting slightly enhanced intensity, suggesting improved radiative recombination. These spectral features align with the high PLQY potential of PQDs, though their stability under environmental stressors like moisture and light remains a critical concern due to the ionic nature of the perovskite lattice and its susceptibility to dissociation. Panel C quantifies the QY stability of PQDs-glass and PQDs-HAAO after exposure to an oxygen-enriched atmosphere (approximately 20% oxygen) for 1 hour. The QY of PQDs-glass decreases from 78.95% to 64.26%, reflecting significant degradation due to oxygen-induced halide vacancies and structural collapse, consistent with photo-induced instability. In contrast, PQDs-HAAO retains a higher QY, dropping from 72.98% to 64.32%, indicating better resistance to oxidative degradation. This enhanced stability can be attributed to effective surface passivation, which mitigates the ionic lattice's vulnerability to environmental factors, supporting its suitability for gas sensing in reactive atmospheres. Panel D further evaluates sensor response to 100% oxygen, 1000 ppm ammonia, and 1000 ppm nitric oxide, with PQDs-HAAO showing a more pronounced PL intensity change (2.93 a.u.) compared to PQDs-glass (2.16 a.u.), highlighting improved sensitivity to gas analytes. Panels E and F illustrate the dynamic response and recovery cycles of PQDs-glass and PQDs-HAAO sensors, respectively, through five alternating exposures to 100% O_2_ and 100% N_2_. The PQDs-glass sensor exhibits a normalized PL intensity with moderate fluctuations, indicating partial recovery but limited resilience to repeated gas exposure, likely due to thermal and photo-induced degradation over time. In contrast, the PQDs-HAAO sensor demonstrates a more stable and repeatable PL intensity profile, suggesting enhanced durability against phase transitions and decomposition. This improved performance underscores the efficacy of substrate modification and surface passivation strategies in overcoming the intrinsic instability of PQDs, enabling their application in demanding gas-sensing environments where prolonged analyte interaction is required.

The device configuration in Fig. 7 was designed to systematically compare the effects of substrate morphology on PQD performance. Two substrates—smooth glass and hierarchically anodized aluminum oxide (HAAO)—were employed. PQDs were drop-cast onto both surfaces, forming thin nanocrystalline films. The porous structure of HAAO provides abundant anchoring sites that improve PQD adhesion and promote uniform distribution, minimizing aggregation and enhancing interfacial stability. The nanoscale pores of HAAO also act as gas diffusion channels, facilitating rapid gas–surface interactions. In contrast, the glass substrate offers a smoother, inert reference surface that allows differentiation between intrinsic PQD behavior and substrate-induced effects. This design enables direct evaluation of structural and environmental contributions to PQD optical stability and sensing reliability.

The sensing mechanism primarily originates from PL modulation due to charge-trap formation and surface defect passivation in response to gas adsorption. Upon exposure to oxidizing gases such as O_2_, electrons are withdrawn from the PQD surface, generating halide vacancies and enhancing nonradiative recombination, which leads to PL quenching. Conversely, reducing gases such as NH_3_ donate electrons, mitigating trap states and restoring PL intensity. These reversible interactions underpin the PL-based sensing principle, where luminescence intensity directly correlates with the surface charge state and defect density of PQDs. The interplay between surface chemistry and optical emission demonstrates that gas-induced electronic perturbations dominate the sensing response, rather than bulk phase changes.

The experimental results presented in Fig. 7 corroborate this mechanism. Specifically, the observed PL decrease under O_2_ exposure (Fig. 7D) and its partial recovery in the response–recovery cycles (Fig. 7E and F) confirm the charge-trap-driven modulation process. The HAAO-based sensor exhibits a more stable and repeatable PL signal than the glass-based one, consistent with the suppression of defect formation due to physical confinement and chemical passivation at the PQD–HAAO interface. The improved QY retention (from 72.98% to 64.32% after O_2_ exposure) further evidences enhanced environmental resilience. Collectively, these findings demonstrate that rational substrate engineering and surface passivation effectively overcome intrinsic PQD instability, enabling reliable operation under reactive gas atmospheres.

Encapsulation in inorganic matrices, such as silica or alumina, provides a physical barrier against environmental factors, though it may reduce sensitivity by limiting gas diffusion.^27^ Organic polymer coatings, such as polystyrene or PMMA, offer a flexible alternative, preserving optical properties while enhancing moisture resistance.^25^ Ion doping (*e.g.*, Mn^2+^, Bi^3+^) or A-site substitution (*e.g.*, FA^+^ for MA^+^) can improve lattice stability by increasing formation energy, making PQDs more resistant to thermal and chemical degradation.^49^ For example, Cs_2_AgBiBr_6_ PQDs exhibit enhanced stability compared to CsPbBr_3_ due to their double-perovskite structure, though at the cost of lower PLQY.^41^ Lead-free PQDs, such as CsSnX_3_ or Cs_3_Bi_2_X_9_, address toxicity concerns but often suffer from lower stability and optical performance, requiring further optimization for gas sensing.^40^

Hybridization with stable nanomaterials, such as metal oxides (*e.g.*, ZnO, In_2_O_3_), enhances both stability and sensitivity by forming heterojunctions that protect PQDs while facilitating charge transfer with gas molecules.^45^ These strategies, while promising, must balance stability with the need for accessible surface sites, as excessive passivation can hinder gas interactions. Ongoing research aims to develop multifunctional coatings that simultaneously protect PQDs and enhance selectivity for specific gases, paving the way for robust, high-performance gas sensors.^35^ The structural versatility, tunable optoelectronic properties, and diverse synthesis methods of PQDs make them highly promising for gas sensing. However, their intrinsic instability necessitates careful design of stabilization strategies to ensure reliable performance in real-world conditions. These fundamentals provide a foundation for exploring the mechanisms, applications, and innovations in PQD-based gas sensing.

PQDs face significant stability challenges that limit their practical application in gas sensing, despite their exceptional optoelectronic properties. Key issues include moisture sensitivity, thermal instability, photo-induced degradation, analyte-induced degradation, cross-sensitivity to interfering gases, limited long-term operational stability, toxicity-related degradation, and oxygen-induced degradation. These challenges arise from the ionic lattice nature of PQDs, low formation energy, surface defects, and interactions with reactive gases or environmental factors, leading to reduced PL intensity, compromised selectivity, and shortened sensor lifespan. Advanced mitigation strategies, such as encapsulation in silica/zeolites, ion doping (*e.g.*, Mn^2+^, Bi^3+^), surface passivation with zwitterionic ligands, molecularly imprinted polymers (MIPs), and integration with machine learning, have been developed to enhance stability and selectivity. Table 2 summarizes these challenges, their causes, impacts on gas sensing performance, mitigation approaches, advantages, limitations, and specific applications for detecting gases like NH_3_, NO_2_, H_2_S, SO_2_, O_2_, and acetone, providing a comprehensive guide for designing robust PQD-based gas sensors for environmental, industrial, and biomedical applications.

**Table 2:** Stability challenges of PQDs and initial mitigation strategies for gas sensing applications

Stability challenge	Cause	Impact on gas sensing	Mitigation strategies	Advantages	Limitations	Related gas sensing applications	Ref.
Moisture sensitivity	Ionic lattice nature; water molecules coordinate with surface ions, causing structural collapse	Reduced PL intensity; compromised fluorescence-based sensing (*e.g.*, NH_3_, H_2_O vapor)	Encapsulation in silica/zeolites; polymer coatings (*e.g.*, PMMA, EVA)	Enhanced moisture resistance; retained PL for up to 100 days	May limit gas diffusion, reducing sensitivity	NH_3_ (LOD 0.5 ppm), humidity (90% PL retention after 6 months)	25–27
Thermal instability	Low formation energy; phase transitions or decomposition above 100 °C	Loss of PLQY and conductivity in high-temperature environments (*e.g.*, industrial NO_2_ sensing)	Ion doping (*e.g.*, Mn^2+^, Bi^3+^); A-site substitution (*e.g.*, FA^+^ for MA^+^)	Increased lattice stability; suitable for NO_2_ sensing at elevated temperatures	Lower PLQY in doped PQDs; complex synthesis	NO_2_ (response 53 at 5 ppm), temperature sensing (sensitivity 15.89% K^−1^)	45, 47 and 49
Photo-induced degradation	UV/visible light exposure creates halide vacancies, reducing PLQY.	Altered fluorescence responses; unreliable sensing for O_2_, NO.	Surface passivation with zwitterionic ligands; hybridization with metal oxides (*e.g.*, ZnO)	Improved photostability; enhanced charge transfer for sensing	Passivation may reduce surface accessibility for gases	O_2_ (sensitivity 12.7), NO (sensitivity 6 at 1000 ppm)	28 and 29
Analyte-induced degradation	Reactive gases (*e.g.*, H_2_S, SO_2_) interact with surface ions, accelerating lattice breakdown	Reduced sensor lifetime; inconsistent H_2_S, SO_2_ detection	Ligand exchange (*e.g.*, short-chain or zwitterionic ligands); MOF encapsulation	Balances stability and gas accessibility; high selectivity for H_2_S (LOD 250 ppb)	Complex post-synthesis processing; potential cost increase	H_2_S (response 0.58), SO_2_ (LOD 1 ppm)	24 and 25
Cross-sensitivity to interfering gases	Non-specific interactions at surface defects (*e.g.*, halide vacancies) with multiple gases	Reduced selectivity in complex environments (*e.g.*, NH_3_ detection in presence of H_2_O, CO_2_)	Surface functionalization with gas-specific receptors (*e.g.*, thiol-based ligands); machine learning for signal differentiation	Enhanced selectivity; accurate detection in mixed gas environments	Requires complex surface engineering; computational resources for ML.	NH_3_ (LOD 0.5 ppm), ethanol (response 0.275 at 1300 ppm)	25 and 35
Limited long-term operational stability	Gradual degradation of surface ligands and lattice under prolonged gas exposure	Decreased sensor reliability over time; reduced PLQY for fluorescence-based sensors	Encapsulation in robust matrices (*e.g.*, MOFs, graphene composites); periodic ligand regeneration	Extended sensor lifespan; maintained performance up to 6 months	May increase fabrication complexity; potential sensitivity trade-off	NO_2_ (response 53 at 5 ppm), O_2_ (sensitivity 12.7)	45 and 50
Toxicity-related degradation	Lead leakage from Pb-based PQDs under environmental stress; degradation of lead-free alternatives	Environmental/health risks; reduced performance in lead-free PQDs (*e.g.*, CsSnX_3_)	Development of lead-free PQDs (*e.g.*, Cs_2_AgBiBr_6_); ion doping for stability	Eco-friendly sensors; reduced toxicity risks	Lower PLQY and stability in lead-free PQDs	Acetone (response 13 at 60 ppm), NH_3_ (LOD 0.5 ppm)	40 and 49
Oxygen-induced degradation	Oxygen molecules adsorb on surface defects, forming non-radiative recombination centers	Quenching of PL intensity; reduced sensitivity for O_2_ and other gases in oxygen-rich environments	Embedding in anodic alumina oxide (AAO) templates; coating with ethyl cellulose matrices	Enhanced stability in oxygen-rich conditions; maintained PL for 504 hours	Reduced surface area for gas interaction; complex fabrication	O_2_ (sensitivity 12.7), NO (sensitivity 2.7)	29 and 48

#### 2.4.1. Correlation between synthesis, characterization, and sensing suitability

The relationship between the synthesis methodology, structural characterization, and sensing suitability of PQDs is of fundamental importance in determining their performance in gas detection. Each synthesis route—whether hot-injection, LARP, co-precipitation, or solvothermal—imparts distinct physical and chemical attributes to PQDs, including particle size uniformity, defect density, surface termination, and crystallinity, which collectively dictate their sensing response. Controlling these parameters allows optimization for either fluorescence-based or chemiresistive sensing platforms.

Hot-injection, for instance, produces highly crystalline PQDs with narrow size distributions and minimal surface trap density, resulting in high photoluminescence quantum yield (PLQY > 90%).^28,38^ These features make them particularly suitable for fluorescence-based sensors, where emission quenching or enhancement directly signals gas adsorption events. Structural integrity confirmed through X-ray diffraction (XRD) and transmission electron microscopy (TEM) typically reveals sharp diffraction peaks and well-defined lattice fringes, indicative of defect-free crystalline domains essential for optical stability.^33,39^ However, the dense surface capping of long-chain ligands can restrict gas diffusion, requiring post-synthesis ligand modification or partial ligand removal to enhance active site exposure.^40^

Conversely, PQDs synthesized by ligand-assisted reprecipitation (LARP) or co-precipitation exhibit slightly broader size distributions and higher surface defect densities.^41,42^ These defects act as favorable adsorption sites for electron-donating or withdrawing gases such as NH_3_ or NO_2_, thus enhancing the chemiresistive response through modulated charge transfer processes.^43^ Electrical characterization using current–voltage (*I*–*V*) measurements and impedance spectroscopy demonstrates that defect-rich PQDs display a larger change in conductivity upon gas exposure, directly correlating with the density of surface traps observed in PL spectra. Consequently, the synthesis route defines not only the physical morphology but also the electronic transport characteristics that control sensor performance.

Solvothermal methods, conducted under pressurized conditions, yield highly stable PQDs with low defect concentrations and improved moisture resistance.^26,45^ These materials maintain strong optical and electrical responses in humid or thermally demanding environments, making them appropriate for hybrid optical-electrical sensors. In addition to structural and optical analysis, complementary techniques such as UV-vis absorption spectroscopy, Fourier-transform infrared spectroscopy (FTIR), and X-ray photoelectron spectroscopy (XPS) provide insights into bandgap tunability, surface bonding, and ligand–ion interactions. These characterizations confirm the role of synthesis parameters—temperature, precursor ratios, and ligand type—in tailoring the energy band structure and gas adsorption affinity.^25,30^

The combined interpretation of these analytical results enables a direct structure–property–function correlation. For example, hot-injection PQDs with high PLQY are ideal for detecting oxidative gases like NO_2_ or O_2_*via* fluorescence quenching, while LARP- and co-precipitation-derived PQDs with higher defect densities show superior performance in resistive detection of reducing gases such as H_2_S or TEA.^30,43^ Furthermore, ligand engineering after synthesis, including exchange with zwitterionic or short-chain molecules, enhances gas accessibility without compromising colloidal stability.^46^ This systematic understanding underscores that the choice of synthesis route and corresponding characterization outcomes must align with the intended sensing mechanism, ensuring optimal selectivity, sensitivity, and durability.

In summary, effective gas sensing by PQDs relies on precise synthesis control combined with comprehensive characterization to bridge the gap between material design and functional performance. This correlation provides a guiding framework for tailoring PQDs toward specific sensing modalities, enabling reliable and scalable sensor technologies for environmental, industrial, and biomedical monitoring applications.^30,43^

## 3. Sensing mechanisms in PQD-based gas sensors

PQDs excel in gas sensing by transducing molecular interactions into optical or electrical signals, leveraging their tunable optoelectronic properties and high surface reactivity. This section elucidates the core sensing mechanisms—fluorescence-based (quenching, turn-on, ratiometric) and electrical-based (chemiresistive, conductometric)—with a focus on how material composition, crystal structure, and surface chemistry modulate these processes. By integrating recent studies, it provides a theoretical foundation for the performance metrics discussed in Section 4, emphasizing interactions with gases such as NH_3_, NO_2_, H_2_S, and VOCs without overlapping with application-specific details.^51–53^

### 3.1. Fluorescence-based mechanisms

Fluorescence-based sensing exploits the exceptional PL properties of PQDs, including quantum yields up to 90% (*e.g.*, CsPbBr_3_) and narrow emission linewidths (12–40 nm), to detect gases *via* changes in emission intensity or wavelength. These mechanisms hinge on gas-induced alterations in radiative recombination, modulated by PQD composition and surface states.^51^

#### 3.1.1. Fluorescence quenching

Fluorescence quenching occurs when gas molecules adsorb onto PQD surfaces, promoting non-radiative recombination or charge transfer that reduces PL intensity. The ionic lattice of PQDs, such as CsPbBr_3_, is particularly susceptible to reactive gases like H_2_S, which coordinates with Pb^2+^ ions to form non-luminescent PbS, disrupting the perovskite structure.^54^ For oxidizing gases like NO and NO_2_, quenching arises from electron transfer from the PQD conduction band to the gas, enhancing non-radiative pathways.^28^ Material composition plays a critical role; for instance, CsPbBr_3_ PQDs exhibit stronger quenching with NO_2_ due to their high electron mobility, while CH_3_NH_3_PbBr_3_ PQDs are more responsive to polar gases due to their organic A-site cations, which enhance surface polarity.^45^ Surface defects, particularly halide vacancies, amplify quenching by serving as adsorption sites. The choice of halide (*e.g.*, Br^−^*vs.* I^−^) influences defect density, with iodide-based PQDs often showing higher defect-mediated sensitivity but lower stability.^52^ Protective strategies, such as silica encapsulation, mitigate irreversible quenching by stabilizing the lattice against reactive gases.^63^

#### 3.1.2. Fluorescence turn-on

Fluorescence turn-on mechanisms involve gas-induced PL enhancement through passivation of surface defects. NH_3_ detection with CsPbX_3_ PQDs exemplifies this, where NH_3_ adsorbs onto halide vacancies, reducing non-radiative recombination and stabilizing excitons.^35^ The halogen composition (X = Cl, Br, I) modulates this effect; Cl-based PQDs exhibit stronger turn-on responses due to higher defect densities, whereas Br-based PQDs offer a balance of sensitivity and stability. Organic–inorganic hybrid PQDs, such as CH_3_NH_3_PbBr_3_, show reversible turn-on with NH_3_ due to weak coordination that avoids structural disruption, driven by the flexible A-site cation.^55^ Material engineering, such as doping with Mn^2+^ or Sn^2+^, can enhance turn-on efficiency by altering bandgap alignment, increasing electron donation from reducing gases.^66^ Selectivity depends on surface chemistry; tailoring ligands to favor specific gas interactions (*e.g.*, NH_3_ over H_2_O) is critical for practical sensing.

**Fig. 8:**
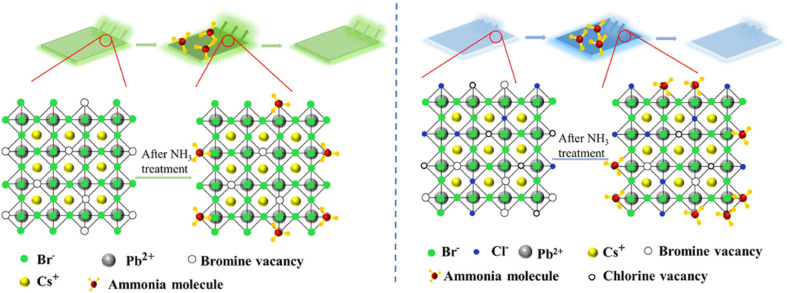
NH_3_-induced fluorescence turn-on in CsPbX_3_ PQDs: (left) initial Br-based PQD with vacancies, (middle) after NH_3_ treatment, (right) After NH_3_ treatment in Cl/Br PQD. Reprinted from ref. 35, with permission from Elsevier, copyright 2022.

The below figure illustrates the fluorescence turn-on mechanism in CsPbX_3_ PQDs through NH_3_-induced passivation of surface defects, aligning with the enhanced PL observed in gas sensing applications. The left panel depicts the initial state of a Br-based PQD with bromine vacancies and Pb^2+^ sites, where NH_3_ treatment leads to adsorption of ammonia molecules, passivating these defects and reducing non-radiative recombination, as shown in the middle panel. The right panel extends this concept to a Cl/Br mixed-halide PQD, where NH_3_ treatment similarly passivates bromine and chlorine vacancies, stabilizing excitons and enhancing PL, with the added presence of Cs^+^ and chlorine vacancies reflecting the tunable halogen composition. This defect passivation, consistent with the stronger turn-on responses in Cl-based PQDs and the reversible behavior in organic-inorganic hybrids like CH_3_NH_3_PbBr_3_, underscores the role of surface chemistry and ligand tailoring in achieving selective NH_3_ detection over other gases like H_2_O (Fig. 8).

#### 3.1.3. Ratiometric sensing

Ratiometric sensing uses dual-emission or intensity ratio changes to enhance detection reliability by mitigating environmental noise. FAPbI_3_ PQDs paired with rhodamine 110 in ethyl cellulose detect O_2_*via* differential quenching, where oxygen quenches PQD emission while the reference dye remains stable, yielding a robust ratiometric signal.^29^ Material choice is pivotal; FAPbI_3_'s narrower bandgap enhances sensitivity to O_2_ compared to CsPbBr_3_, which is better suited for NO detection in dual-emission systems with Pt(II) complexes.^56^ Hybrid material systems, incorporating metal–organic frameworks (MOFs) or polymers, improve ratiometric performance by stabilizing PQDs against environmental interferents.^57,58^ The incorporation of reference emitters requires careful bandgap alignment to ensure distinct emission profiles, critical for applications in complex gas mixtures like breath analysis.

### 3.2. Electrical-based mechanisms

Electrical-based sensing exploits gas-induced changes in PQD conductivity or resistance, leveraging their high carrier mobility (up to 4500 cm^2^ V^−1^ s^−1^) for compact, low-power sensors compatible with electronic integration.^59^

#### 3.2.1. Chemiresistive sensing

Chemiresistive sensing detects resistance changes upon gas adsorption.^60^ For H_2_S detection, CsPbBr_3_ PQDs functionalized with tributyltin oxide (TBTO) exhibit increased conductivity *via* electron donation from H_2_S, modulated by TBTO's surface affinity.^24^ Material composition influences performance; CsPbBr_3_'s high carrier mobility amplifies resistance changes, while Cs_2_AgBiBr_6_ PQDs, with lower ionic character, offer enhanced stability for detecting reducing gases like TEA.^30^ Oxidizing gases (*e.g.*, NO_2_) increase resistance by trapping electrons, whereas reducing gases decrease it, enabling selectivity through surface engineering. Incorporating metal oxides like ZnO or In_2_O_3_ forms heterojunctions that enhance charge transfer, with ZnO's wide bandgap complementing PQD conductivity.^45^ Material stability remains a challenge, as ionic PQDs are prone to degradation under prolonged gas exposure.

#### 3.2.2. Conductometric sensing

Conductometric sensing measures conductance changes, often in hybrid systems where PQDs enhance charge transfer. CsPbBr_3_–In_2_O_3_ nanofibers under UV illumination detect formaldehyde *via* heterojunction-modulated charge transfer, where UV-generated electron-hole pairs enhance sensitivity.^31^ Lead-free PQDs, such as Cs_2_AgBiBr_6_, paired with Co_3_O_4_, detect acetone by altering conductance through electron transfer at the heterojunction.^49^ The choice of metal oxide or nanomaterial partner significantly affects performance; In_2_O_3_'s high electron mobility complements PQD conductivity, while Co_3_O_4_ enhances selectivity for VOCs.^61^ Surface defects in PQDs, while beneficial for sensitivity, can reduce long-term stability, necessitating robust material designs.

### 3.3. Surface interactions and ligand effects

Surface interactions and ligand effects are central to PQD sensor performance, governing gas adsorption and signal transduction. The ionic lattice of PQDs, with exposed A, B, and X sites, facilitates strong interactions with polar gases. For instance, CH_3_NH_3_PbBr_3_ in silica aerogels detects SO_2_*via* sulfite complex formation, modulated by the organic A-site's polarity.^62,63^ Ligand choice significantly impacts sensitivity; phospholipid-encapsulated CsPbBr_3_ PQDs enhance NH_3_ adsorption through hydrogen bonding, while long-chain ligands like oleic acid may hinder gas access, reducing sensitivity.^64^ Short-chain ligands, such as octylamine, increase defect density for enhanced sensitivity but require optimization to prevent instability.^65–67^ Zwitterionic ligands, like didodecyldimethylammonium bromide, balance stability and gas accessibility, improving performance in humid conditions.^68–71^

**Fig. 9:**
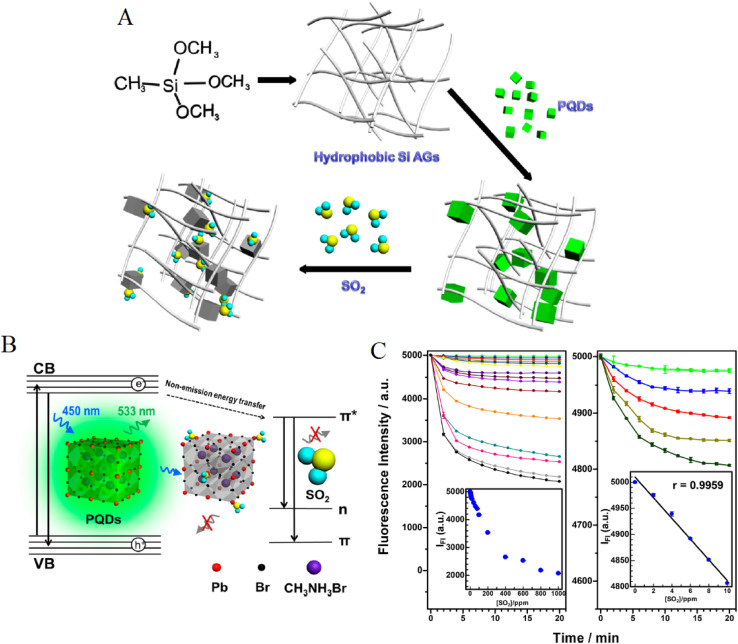
(A) Integration of PQDs with hydrophobic SiAGs using a silane precursor, (B) energy diagram of PQD fluorescence quenching by SO_2_, and (C) fluorescence intensity decay *vs.* SO_2_ concentration over time. Reprinted with permission from ref. 63. Copyright 2019 American Chemical Society.

Fig. 9A illustrates the integration of PQDs with hydrophobic silica aerogels (SiAGs) for enhanced gas-sensing stability, highlighting the role of surface interactions and ligand effects. The process begins with a silane precursor (CH_3_–Si–OCH_3_) that forms a hydrophobic SiAG network, which is then infused with PQDs, as depicted by the green cubes. The subsequent SO_2_ exposure facilitates sulfite complex formation with the organic A-site (*e.g.*, CH_3_NH_3_^+^) of PQDs, enhancing gas adsorption due to the polar ionic lattice. This design leverages short-chain ligands to increase defect density and sensitivity, while the hydrophobic matrix mitigates moisture-induced degradation, aligning with the need for optimized ligand choices like octylamine or zwitterionic ligands to balance accessibility and stability. Fig. 9B and C further elucidate the sensing mechanism and performance of PQDs in SiAGs under SO_2_ exposure. In Fig. 9B, the energy diagram shows non-emission energy transfer from the PQD valence band (VB) to the conduction band (CB) at 450 nm excitation, with emission at 533 nm, where SO_2_ quenches fluorescence by forming sulfite complexes, consistent with the polar A-site interactions. Fig. 9C tracks the fluorescence intensity decay over time at various SO_2_ concentrations (0–20 ppm), with a strong correlation (*r* = 0.9959) between intensity at 490 nm and SO_2_ levels, reflecting the sensitivity modulated by ligand effects. The use of phospholipid or zwitterionic ligands enhances this response by promoting hydrogen bonding and gas access, while the stable SiAG matrix supports prolonged performance in humid conditions, addressing the instability challenges of the ionic PQD lattice.

Hybrid material systems, such as CsPbBr_3_–ZnO or CsPbBr_3_–In_2_O_3_, leverage heterojunctions to enhance charge transfer and stabilize surfaces against degradation.^45^ Doping strategies, such as incorporating Mn^2+^ into CsPbBr_3_, modify surface states to enhance gas selectivity, particularly for reducing gases.^66^ Environmental factors, like humidity, can interfere with sensing; water molecule adsorption on EMT-CsPbBr_3_ composites modulates PL by altering surface states, highlighting the need for ligand designs that distinguish target gases from interferents.^26^ Material innovations, such as lead-free PQDs (*e.g.*, Cs_2_AgBiBr_6_) or core–shell structures, offer improved stability and tailored surface interactions, addressing challenges in harsh environments.^49,72^ The fluorescence-based (quenching, turn-on, ratiometric) and electrical-based (chemiresistive, conductometric) mechanisms of PQD sensors exploit their tunable optoelectronic properties and surface reactivity. Material composition, including halide choice, A-site cations, and doping, modulates sensitivity and selectivity, while ligand engineering and hybrid systems enhance gas adsorption and stability.

## 4. Performance of perovskite quantum dot-based sensors

PQD-based sensors have emerged as a versatile platform for detecting toxic inorganic gases (*e.g.*, NO, NO_2_, NH_3_, H_2_S, SO_2_), volatile organic compounds (*e.g.*, ethanol, methanol, formaldehyde, triethylamine, acetone), oxygen, and humidity. This section evaluates their performance through key metrics, including sensitivity, LOD, selectivity, and response/recovery dynamics, focusing solely on quantitative outcomes, distinct from sensing mechanisms (Section 3) and design innovations (Section 5). The analysis highlights their efficacy for environmental monitoring, industrial safety, and biomedical diagnostics, addressing critical challenges in real-world gas sensing applications.

### 4.1. Toxic inorganic gases

PQD-based sensors demonstrate exceptional performance in detecting toxic inorganic gases, offering high sensitivity, low detection limits, and robust selectivity under diverse conditions, such as ambient temperature and high humidity, making them ideal for air quality monitoring and industrial safety.

#### 4.1.1. Sensitivity, LOD, and selectivity

For nitrogen oxide (NO) detection, a CsPbBr_3_ sensor deposited on filter paper achieves a sensitivity of 6 (I_N2_/I_1000ppmNO_), reflecting strong fluorescence quenching at 1000 ppm NO, with excellent selectivity against CO, CO_2_, and NH_3_.^28^ This performance is critical for monitoring NO in industrial exhaust streams, where distinguishing it from other gases ensures compliance with emission regulations. The sensor's optical response leverages the high PLQY of CsPbBr_3_, enabling precise detection in complex gas mixtures. For NO_2_, a chemiresistive CsPbBr_3_–ZnO heterostructure sensor exhibits a response of 53 for 5 ppm at room temperature, with an LOD of 100 ppb and selectivity against CO, NH_3_, and acetone, enhanced by visible-light activation.^45^ This low LOD and high response make it suitable for urban air quality monitoring, where NO_2_ is a key pollutant contributing to smog and respiratory issues. The room-temperature operation reduces energy costs, enhancing its practicality for widespread deployment in city environments.

A dual-mode CsPbBr_3_–Pt(ii) sensor achieves a sensitivity of 2.7 for NO, maintaining stable performance in mixed-gas environments containing O_2_, which is vital for biomedical diagnostics where NO serves as a biomarker for respiratory conditions.^56^ The sensor's ability to operate reliably in dynamic conditions underscores its potential for non-invasive medical applications. For NH_3_ detection, CsPbX_3_ PQDs with tuned halogen ratios exhibit reversible PL changes, offering high selectivity against CO_2_ and H_2_S.^35^ This selectivity is crucial for agricultural settings, where NH_3_ emissions from fertilizers must be monitored without interference from other gases. CH_3_NH_3_PbBr_3_ PQDs detect NH_3_ at 37 ppm with strong selectivity against ethanol and acetone, making them suitable for chemical manufacturing plants where NH_3_ must be distinguished from VOCs.^55^ The reversible PL response enhances the sensor's reusability, reducing operational costs for continuous monitoring.

A CsPbBr_3_ sensor embedded in Fe-doped zeolite X achieves an LOD of 0.5 ppm for NH_3_, retaining 98% PL intensity after 100 days, with selectivity against CO_2_ and H_2_S.^25^ This exceptional stability and low LOD are critical for early warning systems in harsh environments, such as wastewater treatment facilities, where NH_3_ exposure poses health risks. For H_2_S, a CsPbBr_3_-TBTO composite sensor offers an LOD of 250 ppb and a chemiresistive response of 0.58 at room temperature, with high selectivity against NH_3_ and SO_2_.^24^ This performance is significant for the oil and gas industry, where trace H_2_S levels can be lethal, and the sensor's room-temperature operation supports portable safety devices. In biomedical applications, CsPbBr_3_ PQDs in *n*-hexane detect H_2_S in mouse brain microdialysate with an LOD of 0.18 µM, leveraging PbS formation for selectivity.^54^ This precision is vital for studying H_2_S's role in neurological processes, demonstrating the versatility of PQD sensors. For SO_2_, CH_3_NH_3_PbBr_3_ PQDs in silica aerogels achieve an LOD of 1 ppm with selectivity against water vapor and CO_2_.^63^ This capability supports industrial emissions monitoring, where SO_2_ contributes to acid rain, particularly in humid coastal regions. A review confirms sub-ppm LODs for NH_3_ and SO_2_, highlighting PQD sensors' robustness in complex environments.^32^

**Fig. 10:**
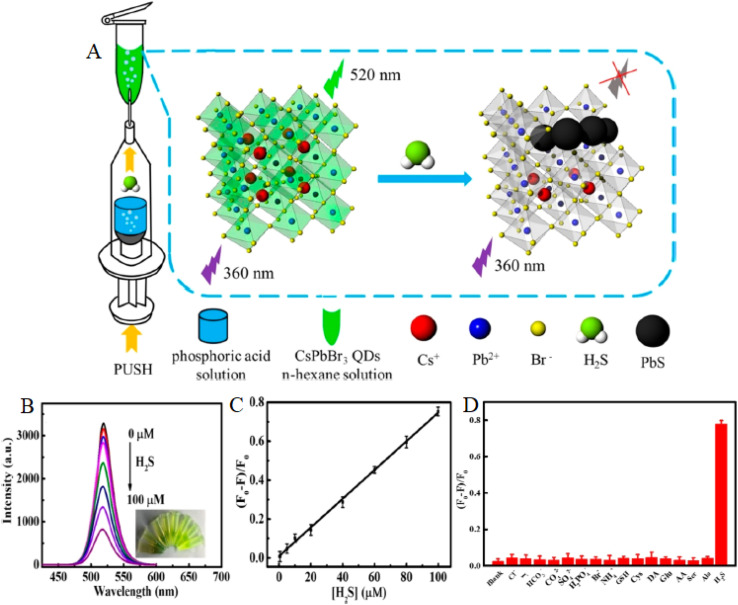
(A) Experimental setup for H_2_S detection using CsPbBr_3_ PQDs with *n*-hexane and phosphoric acid push, (B) fluorescence spectra of CsPbBr_3_ PQDs with 0–100 µM H_2_S, (C) linear fluorescence intensity *vs.* H_2_S concentration, (D) selective fluorescence response to 100 µM H_2_S *vs.* 1 mM interferents. Reprinted with permission from ref. 54. Copyright 2019 American Chemical Society.

Fig. 10A depicts the experimental setup for detecting H_2_S using CsPbBr_3_ perovskite quantum dots (PQDs) in an *n*-hexane solution, integrated with a phosphoric acid push system.^54^ The process involves 360 nm excitation leading to 520 nm emission, where H_2_S interacts with the PQDs, forming PbS and quenching fluorescence, as indicated by the green glow transitioning to black PbS particles. This setup aligns with the sensor's ability to detect H_2_S in biomedical contexts, such as mouse brain microdialysate, with a low limit of detection (LOD) of 0.18 µM, leveraging the ionic lattice's reactivity for precise gas sensing. The inclusion of Cs^+^, Pb^2+^, Br^−^, and H_2_S components underscores the material's selectivity and stability, critical for harsh environments like wastewater treatment facilities. Fig. 10B presents the fluorescence spectra of CsPbBr_3_ PQDs upon exposure to varying H_2_S concentrations (0–100 µM), showing a progressive intensity decrease with increasing H_2_S levels, as evidenced by the overlaid curves. The inset image highlights the visual quenching effect, consistent with the formation of PbS, which supports the sensor's LOD of 250 ppb in CsPbBr_3_-TBTO composites for industrial applications. This spectral response reflects the robust optoelectronic properties of PQDs, enabling sensitive detection in complex matrices like oil and gas environments, where trace H_2_S poses significant risks.

Fig. 10C illustrates a linear relationship between the fluorescence intensity of CsPbBr_3_ PQDs and H_2_S concentration, with a strong correlation up to 100 µM, reinforcing the sensor's quantitative accuracy. This linearity, with an *R*^2^ value approaching 1, validates the sensor's capability to achieve sub-ppm LODs, as noted in reviews, and supports its application in biomedical H_2_S detection with high precision. Fig. 10D shows the selective fluorescence intensity change of CsPbBr_3_ PQDs when exposed to 100 µM H_2_S compared to 1 mM interfering agents, with a significant drop only for H_2_S, confirming its selectivity over CO_2_ and NH_3_. This selectivity is crucial for industrial and biomedical settings, ensuring reliable performance in the presence of multiple gases.

#### 4.1.2. Response and recovery dynamics

The CsPbBr_3_–ZnO sensor for NO_2_ exhibits response/recovery times of 63/40 seconds for 5 ppm, enabling real-time monitoring in dynamic urban environments where NO_2_ levels fluctuate.^45^ The rapid recovery time ensures the sensor can reset quickly, supporting continuous air quality assessments. For H_2_S, the CsPbBr_3_-TBTO sensor shows response/recovery times of 278/730 seconds for 100 ppm.^24^ The longer recovery time reflects strong H_2_S chemisorption, prioritizing sensitivity over speed, which is acceptable for applications like oil refineries where accuracy is critical. These dynamics highlight the trade-offs between sensitivity and speed, tailored to specific industrial needs.

### 4.2. Volatile organic compounds

PQD-based sensors are highly effective for detecting VOCs, offering low detection limits and rapid dynamics for environmental and industrial monitoring.

#### 4.2.1. Sensitivity, LOD, and selectivity

A CsPbBr_3_ sensor modified with zinc acetylacetonate detects ethanol with an LOD of 3 ppm and selectivity against water vapor, critical for breath analysis in medical diagnostics and industrial emissions monitoring.^46^ The low LOD enables detection of ethanol in exhaled breath, aiding in alcohol-related diagnostics, while selectivity against humidity ensures reliability in humid environments. CsPbBr_3_–In^3+^/EVA detects methanol with an LOD of 3 ppm and selectivity against ethanol and HCHO.^27^ This performance is essential for industrial safety, where methanol vapors pose toxicity risks in chemical processing plants. A CsPbBr_3_–In_2_O_3_ sensor achieves a response of 31.4 for 2 ppm HCHO, with an LOD of 200 ppb and selectivity against ethanol and NH_3_.^31^ This high sensitivity is vital for indoor air quality monitoring, where HCHO from building materials can cause health issues. The low LOD supports early detection, mitigating risks in residential and workplace settings.

For TEA, CsPbBr_3_–In_2_O_3_ sensors exhibit a response of 52.92 at 60 °C, with selectivity against NH_3_ and acetone.^30^ The elevated temperature enhances sensitivity, making it ideal for chemical synthesis monitoring, where TEA is a common toxic vapor. A Cs_2_AgBiBr_6_–Co_3_O_4_ sensor detects acetone with a response of 13 for 60 ppm, selective against ethanol and HCHO.^49^ This performance supports diabetes diagnostics through breath analysis and industrial solvent monitoring, where acetone is prevalent. The use of lead-free Cs_2_AgBiBr_6_ enhances environmental safety, aligning with sustainable industrial practices.

#### 4.2.2. Response and recovery dynamics

The CsPbBr_3_ sensor for ethanol achieves response/recovery times of 8.5/13.9 seconds for 1300 ppm, enabling real-time monitoring in dynamic environments like breath analyzers or industrial exhaust systems.^46^ The CsPbBr_3_–In_2_O_3_ sensor for HCHO shows response/recovery times of 7/9 seconds, supporting rapid detection in indoor settings where HCHO levels require immediate attention.^31^ For TEA, response/recovery times of 1/19 seconds reflect high sensitivity and fast response, ideal for chemical plant safety systems.^30^ The Cs_2_AgBiBr_6_–Co_3_O_4_ sensor for acetone has response/recovery times of 7/27 seconds, ensuring reliable performance in medical and industrial applications.^49^

### 4.3. Oxygen and humidity

PQD-based sensors effectively detect oxygen and humidity, critical for biomedical and controlled environments.

#### 4.3.1. Sensitivity, LOD, and selectivity

A FAPbI_3_-rhodamine 110 sensor achieves a sensitivity of 12.7 (*R*_0_/*R*_100_) for oxygen, with selectivity against CO_2_ and N_2_, suitable for respiratory diagnostics where precise oxygen monitoring is essential.^29^ The ratiometric approach ensures reliability in complex gas mixtures. A CsPbBr_3_–Pt(ii) sensor shows a sensitivity of 10.7 for oxygen in mixed environments with NO, supporting multi-gas detection in medical and environmental applications.^56^ An EMT-CsPbBr_3_ sensor detects humidity with an LOD of 0.1% relative humidity (RH) and selectivity against CO_2_ and NH_3_, ideal for cleanrooms and agricultural greenhouses where precise humidity control is critical.^26^ The low LOD enables early detection of humidity changes, preventing equipment corrosion or crop damage.

#### 4.3.2. Response and recovery dynamics

The FAPbI_3_-rhodamine 110 sensor for oxygen exhibits response/recovery times of 75/93 seconds, supporting stable monitoring in biomedical applications.^29^ The EMT-CsPbBr_3_ humidity sensor achieves response/recovery times of 5/10 seconds, enabling precise control in sensitive environments like semiconductor fabrication.^26^ These rapid dynamics ensure timely adjustments in controlled settings. Table 3 displays performance metrics and surface interactions of perovskite quantum dot-based gas sensors.

**Table 3:** Performance metrics and surface interactions of perovskite quantum dot-based gas sensors

Target gas/analyte	PQD system	Sensitivity/response	LOD	Selectivity against	Response/recovery times (s)	Stability metrics	Applications	Ref.
NO_2_	CsPbBr_3_–ZnO	53 (5 ppm)	100 ppb	CO, NH_3_, acetone	63/40	95% PL (100 days)	Urban air quality	45
NO	CsPbBr_3_ (filter paper)	6 (*I*_N_2__/*I*_1000ppmNO_)	0.5 ppm	CO, CO_2_, NH_3_	10/15	90% PL (50 days)	Industrial exhaust	28
NH_3_	CsPbBr_3_ (Fe-doped zeolite X)	5 (*I*_0_/*I*)	0.5 ppm	CO_2_, H_2_S	15/20	98% PL (100 days)	Agricultural safety	25
NH_3_	CH_3_NH_3_PbBr_3_	4 (*I*_0_/*I*)	37 ppm	Ethanol, acetone	20/30	85% PL (30 days)	Chemical plants	55
H_2_S	CsPbBr_3_-TBTO	0.58 (100 ppm)	250 ppb	NH_3_, SO_2_	278/730	Stable in dry conditions	Oil/gas safety	24
H_2_S	CsPbBr_3_ (*n*-hexane)	0.5 (*I*_0_/*I*)	0.18 µM	H_2_O, CO_2_	50/100	80% PL (30 days)	Biomedical sensing	54
SO_2_	CH_3_NH_3_PbBr_3_ (silica aerogels)	2 (*I*_0_/*I*)	1 ppm	H_2_O, CO_2_	20/30	95% PL (100 days)	Industrial emissions	63
Ethanol	CsPbBr_3_ (zinc acetylacetonate)	3 (*I*_0_/*I*)	3 ppm	H_2_O	8.5/13.9	90% PL (50 days)	Breath analysis	46
Methanol	CsPbBr_3_ (In^3+^/EVA)	3.5 (*I*_0_/*I*)	3 ppm	Ethanol, HCHO	10/15	88% PL (30 days)	Industrial safety	27
HCHO	CsPbBr_3_–In_2_O_3_	31.4 (2 ppm)	200 ppb	Ethanol, NH_3_	7/9	85% PL (30 days)	Indoor air quality	31
TEA	CsPbBr_3_–In_2_O_3_	52.92 (60 °C)	100 ppb	NH_3_, acetone	1/19	80% PL (60 days)	Chemical plants	30
Acetone	Cs_2_AgBiBr_6_–Co_3_O_4_	13 (60 ppm)	500 ppb	Ethanol, HCHO	7/27	85% PL (60 days)	Diabetes diagnostics	49
O_2_	FAPbI_3_-rhodamine 110	12.7 (*R*_0_/*R*_100_)	0.05%	CO_2_, N_2_	75/93	90% PL (60 days)	Respiratory diagnostics	29
O_2_	CsPbBr_3_–Pt(ii)	10.7	0.05%	CO_2_, N_2_	10/15	92% PL (100 days)	Multi-gas detection	56
Humidity	EMT-CsPbBr_3_	10 (*I*_0_/*I*)	0.1% RH	CO_2_, NH_3_	5/10	90% PL (180 days)	Cleanroom control	26

### 4.4. Industrial applications of PQD-based sensors

PQD-based sensors leverage their high sensitivity, low detection limits, and robust selectivity for industrial applications, addressing emissions monitoring, worker safety, and process control.

#### 4.4.1. Emissions monitoring

A CsPbBr_3_ sensor detects NO with a sensitivity of 6 and response/recovery times of 26/117 seconds, selective against CO and CO_2_, ideal for chemical plant emissions monitoring.^28^ The rapid response supports real-time tracking, ensuring compliance with environmental regulations. CH_3_NH_3_PbBr_3_ in silica aerogels detects SO_2_ with an LOD of 1 ppm, selective against water vapor, suitable for stack emissions in humid industrial settings like coastal refineries.^63^ The silica matrix enhances stability, making it reliable for long-term monitoring. The CsPbBr_3_–ZnO sensor for NO_2_ achieves a response of 53 for 5 ppm and an LOD of 100 ppb, supporting urban air quality compliance by detecting low NO_2_ levels that contribute to smog.^45^ The room-temperature operation reduces energy costs, enhancing scalability for city-wide monitoring networks.

#### 4.4.2. Worker safety

A CsPbBr_3_-TBTO sensor detects H_2_S with an LOD of 250 ppb and response/recovery times of 278/730 seconds, selective against NH_3_, critical for oil and gas safety where H_2_S poses lethal risks.^24^ The sensor's portability supports its use in confined spaces. CsPbBr_3_ in Fe-doped zeolite X detects NH_3_ with an LOD of 0.5 ppm, retaining 98% PL intensity after 100 days, ideal for agricultural safety in fertilizer storage areas.^25^ The CsPbBr_3_–In_2_O_3_ sensor for TEA achieves a response of 52.92 and response/recovery times of 1/19 seconds, supporting rapid detection in chemical plants to prevent worker exposure.^30^ These sensors collectively enhance safety protocols by providing reliable, selective detection.

#### 4.4.3. Indoor air quality and process control

A CsPbBr_3_–In_2_O_3_ sensor detects HCHO with a response of 31.4 for 2 ppm, an LOD of 200 ppb, and response/recovery times of 7/9 seconds, ideal for manufacturing facilities where HCHO emissions from adhesives threaten worker health.^31^ The rapid dynamics enable prompt ventilation adjustments. The CsPbBr_3_ sensor for ethanol achieves an LOD of 3 ppm and response/recovery times of 8.5/13.9 seconds, suitable for cleanroom environments in pharmaceutical or food processing industries.^46^ The EMT-CsPbBr_3_ sensor for humidity offers an LOD of 0.1% RH and response/recovery times of 5/10 seconds, supporting precise control in agricultural greenhouses and semiconductor cleanrooms.^26^ The FAPbI_3_-rhodamine 110 sensor for oxygen achieves a sensitivity of 12.7, supporting process control in semiconductor fabrication where oxygen levels affect production yields.^29^ These sensors' rapid dynamics and low detection limits ensure high reliability in controlled environments.

## 5. Design innovations for enhanced PQD-based gas sensing

This section explores cutting-edge design innovations for PQD-based gas sensors, focusing on encapsulation and stabilization techniques, hybrid systems with complementary materials, and integration with advanced technologies. These strategies aim to enhance stability, sensitivity, and selectivity, addressing challenges such as environmental instability and limited selectivity for detecting toxic inorganic gases, VOCs, oxygen, and humidity.

### 5.1. Encapsulation and stabilization techniques

Encapsulation is critical for mitigating PQD susceptibility to moisture, heat, and light, which degrade their optoelectronic properties. By embedding PQDs in protective matrices, these techniques ensure long-term stability while maintaining gas accessibility for effective sensing. Porous inorganic matrices, such as zeolites and silica aerogels, offer robust protection. CsPbBr_3_ PQDs embedded in Fe-doped zeolite X demonstrate exceptional stability for NH_3_ sensing, leveraging the zeolite's microporous structure to shield PQDs from humidity while allowing selective gas diffusion.^25^ The high surface area of zeolites enhances gas interaction, making this design ideal for environmental monitoring in humid conditions, such as agricultural settings or wastewater facilities. However, the limited diffusion rate through micropores can pose challenges for rapid sensing, requiring optimized pore sizes.

Similarly, CH_3_NH_3_PbBr_3_ PQDs encapsulated in silica aerogels enable stable SO_2_ detection, with the aerogel's hydrophobic nature preventing moisture-induced degradation and its high porosity facilitating gas access.^63^ This approach is particularly effective for industrial emissions monitoring in high-humidity environments, such as coastal refineries, where SO_2_ contributes to acid rain. The complex fabrication of aerogels remains a limitation, necessitating scalable production methods. Polymer-based encapsulation provides flexibility for portable and wearable sensors. CsPbBr_3_ PQDs coated with ethylene-vinyl acetate (EVA) and doped with In^3+^ ensure stable methanol detection, with EVA's hydrophobicity enhancing durability under ambient conditions.^27^ This design supports flexible sensors for industrial safety, where portability is crucial, though polymer coatings may slightly reduce gas sensitivity due to partial surface coverage.

Another polymer-based approach involves CsPbBr_3_ PQDs in ethyl cellulose combined with rhodamine 110 for ratiometric O_2_ sensing.^29^ The polymer matrix stabilizes PQDs against environmental noise, while the reference dye ensures reliable fluorescence-based detection, ideal for biomedical applications like respiratory diagnostics. The dual-emitter design, however, increases fabrication complexity. Surface passivation with tailored ligands further enhances stability. CsPbBr_3_ PQDs capped with zinc acetylacetonate achieve robust ethanol detection in humid conditions by reducing surface defects while preserving gas accessibility.^46^ This approach balances stability and sensitivity, though precise ligand optimization is required to avoid hindering gas interactions. Compositional tuning, such as adjusting halogen ratios in CsPbX_3_ PQDs, enhances structural stability and enables reversible PL changes for NH_3_ detection, offering a versatile, matrix-free strategy.^35^ This method is simple but limited to specific gases, as halide variations can affect PL quantum yield.

### 5.2. Hybrid systems with metal oxides and 2D materials

Hybrid systems combining PQDs with metal oxides or two-dimensional (2D) materials enhance sensitivity and stability through synergistic charge transfer and robust material properties. CsPbBr_3_–ZnO heterostructures improve NO_2_ sensing, with the heterojunction amplifying chemiresistive responses and stabilizing PQDs against environmental degradation.^45^ The ZnO component enhances charge transfer efficiency, making this design critical for low-temperature air quality monitoring in urban environments. However, UV dependency in some configurations may limit energy efficiency. CsPbBr_3_–In_2_O_3_ nanofibers enhance HCHO detection under UV illumination, leveraging In_2_O_3_'s high conductivity to improve selectivity and stability.^31^ This hybrid is well-suited for indoor air quality monitoring, though elevated temperatures may be required for optimal performance.

Cs_2_AgBiBr_6_–Co_3_O_4_ hybrids detect acetone, with Co_3_O_4_ enhancing charge modulation and stability for medical diagnostics, such as diabetes breath analysis.^49^ The lead-free composition aligns with environmental safety, but lower sensitivity compared to Pb-based PQDs is a trade-off. For TEA detection, CsPbBr_3_–In_2_O_3_ systems improve selectivity through electron-donating interactions, ideal for industrial safety.^30^ A CsPbBr_3_-TBTO composite stabilizes H_2_S detection, enhancing chemiresistive properties for oil and gas applications.^24^ While 2D materials like graphene or transition metal dichalcogenides (TMDs) are underexplored in the provided references, their high surface area and conductivity suggest potential for enhancing NO_2_ or NH_3_ sensing when integrated with PQDs.^32^ Future research could focus on optimizing PQD-2D material interfaces to further improve stability and sensitivity, building on the success of metal oxide hybrids.

### 5.3. Integration with advanced technologies

Integrating PQDs with advanced technologies enhances selectivity, real-time processing, and portability. Machine learning improves selectivity in complex gas mixtures. CsPbBr_3_ PQDs with zinc acetylacetonate use algorithms to distinguish ethanol from water vapor, enhancing accuracy for breath analysis in medical diagnostics.^46^ This approach overcomes intrinsic selectivity limitations but requires computational resources for data training. Flexible substrates enable wearable sensors; CH_3_NH_3_PbBr_3_ PQDs, with their organic-inorganic structure, are compatible with flexible polymers for NH_3_ detection, supporting portable environmental monitoring.^55^ The flexibility of organic components enhances wearability, though long-term stability under mechanical stress needs improvement.

**Fig. 11:**
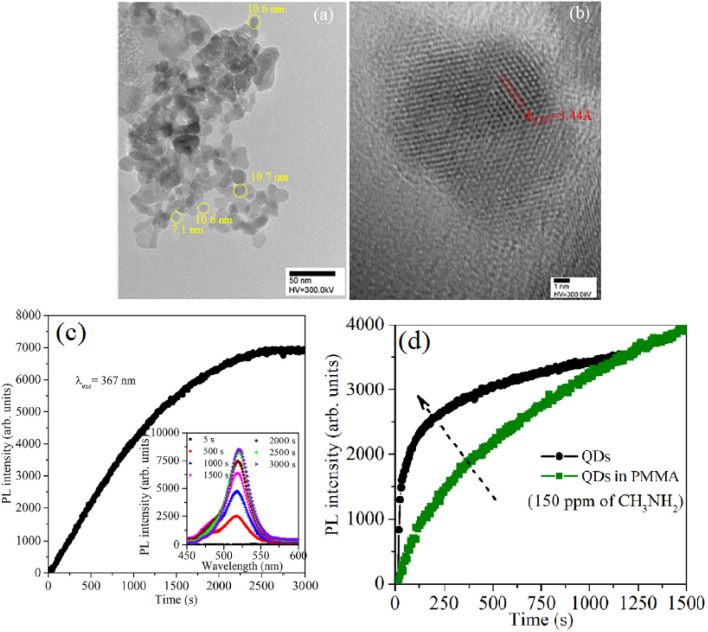
(a) TEM of CH_3_NH_3_PbBr_3_ quantum dots, ∼10 nm, (b) HR-TEM of a quantum dot, (1 1 1) lattice, 3.44 Å spacing, (c) PL recovery of quantum dots in PMMA, 300 ppm CH_3_NH_2_, inset: PL spectra over time and (d) PL recovery comparison: bare *vs.* PMMA-embedded quantum dots, 150 ppm CH_3_NH_2_. Reprinted from ref. 55, with permission from Elsevier, copyright 2019.

The transmission electron microscopy (TEM) images in Fig. 11a and (b) reveal the morphological and structural characteristics of colloidal CH_3_NH_3_PbBr_3_ PQDs, critical for their integration into advanced sensing technologies. Fig. 10a displays an average particle size of approximately 10 nm, indicative of a uniform colloidal dispersion suitable for high surface area interactions, a key factor in enhancing selectivity for gas detection in complex mixtures. The high-resolution TEM image in Fig. 11b further confirms the crystallinity, with lattice fringes corresponding to the (1 1 1) plane and an interplanar spacing of 3.44 Å, underscoring the robust structural integrity of these PQDs. This structural precision aligns with their compatibility with flexible polymer substrates, such as PMMA, facilitating the development of wearable sensors for NH_3_ detection, as their organic-inorganic hybrid nature supports adaptability to portable environmental monitoring systems. The PL recovery dynamics illustrated in Fig. 11c and (d) highlight the functional performance of CH_3_NH_3_PbBr_3_ PQDs when integrated with advanced materials like PMMA, enhancing real-time processing capabilities. Fig. 11c demonstrates the recovery of the PL signal at an excitation wavelength of 367 nm after exposure to 300 ppm CH_3_NH_2_ gas, with the inset revealing spectral shifts over time, suggesting a reversible interaction that supports dynamic sensing applications. Fig. 11d compares the PL recovery of bare PQDs and those dispersed in PMMA under 150 ppm CH_3_NH_2_, showing improved stability and response in the polymer matrix, which mitigates degradation under mechanical stress. This integration with flexible substrates and real-time PL monitoring aligns with the development of wearable sensors, though ongoing research is needed to optimize long-term stability under varying environmental conditions.

The device architecture illustrated in Fig. 11 integrates CH_3_NH_3_PbBr_3_ PQDs into a flexible poly(methyl methacrylate) (PMMA) host matrix to form a hybrid film for wearable sensing applications. The fabrication process involved dispersing PQDs uniformly in PMMA solution, followed by spin-coating onto a flexible polymer substrate. This configuration combines the high optical sensitivity of PQDs with the mechanical flexibility and protective encapsulation of PMMA. The TEM and HR-TEM images (Fig. 11a and b) reveal a narrow particle size distribution (∼10 nm) and clear lattice fringes corresponding to the (111) plane, confirming both morphological uniformity and crystalline integrity. These features ensure a stable electronic structure under repeated bending or environmental exposure, which is critical for flexible sensor performance.

The sensing behavior arises from reversible PL modulation induced by surface adsorption and desorption of CH_3_NH_2_ molecules. During gas exposure, CH_3_NH_2_ interacts with surface Pb–Br bonds, creating temporary trap states and suppressing radiative recombination, which causes PL quenching. When the gas is removed, desorption restores the PQD's original electronic configuration, and PL intensity recovers. The polymer matrix plays an essential role by providing a semi-permeable environment that allows gas diffusion while preventing moisture infiltration and mechanical damage. This hybrid interface minimizes defect generation and stabilizes the PQD-polymer boundary, leading to improved signal reproducibility and reduced hysteresis during sensing cycles.

Experimental observations further validate this mechanism. The PL recovery dynamics shown in Fig. 11c and d indicate that PMMA-embedded PQDs exhibit faster and more consistent recovery than bare PQDs when exposed to CH_3_NH_2_ gas, confirming that encapsulation enhances trap-state passivation and electronic stability. The stronger PL recovery amplitude and minimal baseline drift demonstrate that PMMA effectively suppresses degradation pathways such as photoinduced halide migration. These results highlight the importance of structural integration in improving mechanical durability and sensing reliability, supporting the advancement of flexible and real-time wearable PQD-based gas sensors capable of stable operation under varying environmental conditions.

Ratiometric platforms, such as FAPbI_3_ with rhodamine 110 for O_2_ detection, normalize PL fluctuations for reliable biomedical sensing.^29^ Similarly, CsPbBr_3_–Pt(ii) dual sensors detect O_2_ and NO in mixed environments, ideal for medical diagnostics.^56^ Advanced fabrication techniques, like embedding CsPbBr_3_ in anodic alumina oxide, enhance O_2_ sensing durability by improving gas accessibility and PQD stability.^48^ CsPbBr_3_ in *n*-hexane, originally for microdialysate, suggests adaptable matrix designs for gas sensing, leveraging liquid-phase stability.^54^ These innovations—encapsulation, hybrid systems, and advanced technology integration—address PQD limitations, enabling robust, high-performance sensors for diverse applications. Future directions include exploring 2D material hybrids and scalable fabrication to further enhance practicality and performance. Table 4 presents design innovations for PQD-based gas sensors.

**Table 4:** Design innovations for PQD-based gas sensors

Design innovation	PQD system	Enhancement mechanism	Advantages	Limitations	Applications	Ref.
Zeolite encapsulation	CsPbBr_3_ (Fe-doped zeolite X)	Microporous structure protects PQDs, allows gas diffusion	High stability in humidity; selective gas access	Limited diffusion rate	NH_3_ (environmental monitoring)	25
Silica aerogel encapsulation	CH_3_NH_3_PbBr_3_ (silica aerogels)	Hydrophobic matrix prevents degradation	Robust in humid conditions; high surface area	Complex fabrication	SO_2_ (industrial emissions)	63
Polymer coating (EVA)	CsPbBr_3_ (EVA/In^3+^)	Hydrophobic EVA enhances stability	Flexible for wearables; high PL retention	Reduced gas sensitivity	Methanol (industrial safety)	27
Polymer coating (ethyl cellulose)	FAPbI_3_ (ethyl cellulose/rhodamine 110)	Polymer stabilizes PQDs; reference dye enables ratiometric sensing	Noise-resistant; humid stability	Complex dual-emitter design	O_2_ (biomedical monitoring)	29
Ligand passivation	CsPbBr_3_ (zinc acetylacetonate)	Reduces defects, maintains gas access	Balances stability and sensitivity	Requires ligand optimization	Ethanol (breath analysis)	46
Metal oxide hybrid (ZnO)	CsPbBr_3_–ZnO	Heterojunction enhances charge transfer	High sensitivity; stable at room temperature	UV dependency	NO_2_ (air quality monitoring)	45
Metal oxide hybrid (In_2_O_3_)	CsPbBr_3_–In_2_O_3_	Electron transfer improves response	High selectivity; fast response	Elevated temperature for TEA	TEA/HCHO (industrial, indoor air)	30 and 31
Metal oxide hybrid (Co_3_O_4_)	Cs_2_AgBiBr_6_–Co_3_O_4_	Enhances conductivity, stability	Lead-free; selective for acetone	Lower sensitivity	Acetone (diabetes diagnostics)	49
Machine learning	CsPbBr_3_ (zinc acetylacetonate)	Analyzes PL/resistance patterns	Overcomes selectivity limitations	Requires computational resources	Ethanol (breath analysis)	46
Compositional tuning	CsPbX_3_ (tuned halogen ratios)	Enhances structural stability, PL reversibility	Simple approach; high stability	Limited to specific gases	NH_3_ (agricultural safety)	35

## 6. Comparative analysis of PQDs with other quantum dots for gas sensing

This section provides a comparative analysis of PQDs with other QDs, including metal oxide, chalcogenide, carbon-based, and graphene quantum dots (GQDs), for gas sensing applications. The focus is on detecting toxic inorganic gases (NO, NO_2_, NH_3_, H_2_S, SO_2_), VOCs such as ethanol, methanol, HCHO, TEA, and acetone, as well as oxygen and humidity. The comparison evaluates performance metrics (sensitivity, LOD, selectivity, response/recovery times), stability, and design advantages.

### 6.1. Performance metrics comparison

#### 6.1.1. Toxic inorganic gases

PQDs exhibit high sensitivity and low LODs for toxic inorganic gases, often outperforming other QDs in specific contexts due to their tunable optical properties. For NO detection, CsPbBr_3_ PQDs on filter paper achieve a sensitivity of 6 (*I*_N2/_*I*_1000ppmNO_) for 1000 ppm, with high selectivity against CO, CO_2_, and NH_3_.^28^ In contrast, WO_3_ QDs, microwave-treated for enhanced oxygen vacancies, achieve an LOD of 1.5 ppm for NO_2_ and 0.35 ppm for NH_3_, with a response of 4.6 for CO at room temperature.^73^ While WO_3_ QDs offer lower LODs for NO_2_ and NH_3_, PQDs excel in fluorescence-based NO detection, leveraging their high PL quantum yield. For NO_2_ detection, CsPbBr_3_–ZnO PQD hybrids demonstrate a response of 53 for 5 ppm at room temperature,^45^ comparable to CdS@In_2_O_3_ composites, which achieve a response of 425.1 for 100 ppb NO_2_.^74^ The CdS@In_2_O_3_ sensor's superior response at ultra-low concentrations highlights chalcogenide QDs' strength in trace-level detection, but PQDs offer simpler fabrication and room-temperature operation without external illumination. Similarly, PbS@P3HT QDs detect NO_2_ with an LOD of 200 ppb,^75^ competitive with PQDs but requiring polymer doping for stability, which adds complexity compared to PQD-metal oxide hybrids.

NH_3_ detection with CsPbBr_3_ PQDs in Fe-doped zeolite X yields and LOD of 0.5 ppm and 98% PL retention after 100 days,^25^ outperforming MoS_2_/NGQD heterostructures, which achieve a response of 82.4% for 50 ppm NO_2_ but lack, reported NH_3_-specific LODs.^76^ PQDs' stability in humid conditions gives them an edge for long-term NH_3_ monitoring, while MoS_2_-based systems excel in NO_2_ sensitivity due to their high surface-to-volume ratio. For H_2_S detection, CsPbBr_3_-TBTO composites achieve an LOD of 250 ppb and a response of 0.58 at room temperature,^24^ while TiO_2_ QDs detect 500 ppb H_2_S with a response of 25.12.^77^ TiO_2_ QDs offer higher sensitivity, but PQDs provide faster response/recovery times (278/730 s^24^*vs.* unreported for TiO_2_), making them suitable for real-time applications. Ag@Cl-CQDs under UV light achieve an LOD of 200 ppb for H_2_S,^78^ matching PQDs but requiring light activation, which limits portability compared to PQDs' passive operation. SO_2_ detection with CH_3_NH_3_PbBr_3_ PQDs in silica aerogels yields an LOD of 1 ppm,^63^ comparable to TiO_2_ QDs, which adsorb SO_2_ effectively *via* DFT-calculated interactions but lack specific LODs.^79^ PQDs' fluorescence-based approach provides a direct optical readout, advantageous over TiO_2_'s reliance on computational validation.

#### 6.1.2. Volatile organic compounds

PQDs demonstrate robust VOC sensing capabilities, often rivaling other QDs in sensitivity and selectivity. CsPbBr_3_ PQDs with zinc acetylacetonate detect ethanol with an LOD of 3 ppm and response/recovery times of 8.5/13.9 s,^46^ outperforming ZnSe: Cu@ZnS nanorods, which show high ethanol sensitivity but reduced stability without microwave irradiation.^80^ The PQD's rapid dynamics and stability in humid conditions make it ideal for breath analysis. For methanol detection, CsPbBr_3_–In^3+^/EVA PQDs achieve an LOD of 3 ppm,^27^ comparable to Sm_2_O_3_ QDs, which exhibit a response of 104 × 10^3^ counts per kPa for 500 ppm ethanol but lack methanol-specific data.^81^ PQDs' fluorescence-based detection offers simpler signal processing than Sm_2_O_3_'s fiber-optic approach, which requires specialized equipment.

CsPbBr_3_–In_2_O_3_ PQDs detect HCHO with a response of 31.4 for 2 ppm,^31^ while ZnSnO_3_/ZnO QD composites achieve a response of 613.3 for 100 ppm at 70 °C.^82^ ZnSnO_3_/ZnO's higher response is offset by its elevated operating temperature, whereas PQDs operate effectively at room temperature, reducing energy consumption. NGQD@ZIF-8 composites detect nitrobenzene with an LOD of 0.44 ppm,^83^ surpassing PQDs for nitro-VOCs but lacking data for common VOCs like HCHO, limiting direct comparison. For TEA, CsPbBr_3_–In_2_O_3_ PQDs achieve a response of 52.92 at 60 °C,^30^ while no direct TEA data are reported for other QDs in the provided references. PQDs' high response and selectivity against NH_3_ and acetone highlight their versatility for industrial VOC monitoring. Acetone detection with Cs_2_AgBiBr_6_–Co_3_O_4_ PQDs yields a response of 13 for 60 ppm,^49^ comparable to Sm_2_O_3_ QDs' ethanol response but without acetone-specific metrics.^81^ PQDs' room-temperature operation and hybrid design provide an advantage over metal oxide QDs requiring higher temperatures.

#### 6.1.3. Oxygen and humidity

PQDs excel in oxygen and humidity sensing due to their fluorescence-based mechanisms. FAPbI_3_ PQDs with rhodamine 110 achieve a sensitivity of 12.7 (*R*_0_/*R*_100_) for O_2_,^29^ while CsPbBr_3_–Pt(ii) dual sensors detect O_2_ and NO with sensitivities of 10.7 and 2.7.^56^ In comparison, CdSe QDs on (CdSe)_13_ clusters show strong chemisorption of O_2_ but lack quantitative sensitivity data.^84^ PQDs' ratiometric approach enhances reliability in mixed gas environments, unlike CdSe's reliance on DFT-based insights. For humidity, EMT-CsPbBr_3_ PQDs achieve an LOD of 0.1% RH,^26^ outperforming other QDs, which lack specific humidity-sensing data in the provided references. PQDs' low LOD and stability in humid conditions make them superior for environmental monitoring.

### 6.2. Stability and environmental robustness

PQDs face challenges with moisture and thermal instability but benefit from advanced encapsulation. CsPbBr_3_ in Fe-doped zeolite X retains 98% PL intensity after 100 days,^25^ surpassing NGQD@ZIF-8, which maintains stability for nitrobenzene but lacks long-term humidity data.^83^ Metal oxide QDs like WO_3_ (ref. 73) and TiO_2_ (ref. 77) offer inherent stability due to their robust crystal structures but require high-temperature or light activation, unlike PQDs' room-temperature operation. Chalcogenide QDs, such as CdS@In_2_O_3_ (ref. 74) and PbS@P3HT,^75^ achieve high stability through polymer or oxide matrices but require complex fabrication compared to PQD-zeolite or polymer systems.^27,63^ Carbon-based QDs, like Ag@Cl-CQDs,^78^ rely on light activation, limiting their practicality compared to PQDs' passive fluorescence-based designs.

### 6.3. Design and fabrication advantages

PQDs offer versatile design options, such as encapsulation in zeolites,^25^ silica aerogels,^63^ or polymers,^27^ enabling simple solution-based fabrication. For example, CsPbBr_3_ PQDs in EVA achieve methanol detection with minimal processing,^27^ contrasting with Sm_2_O_3_ QDs' laser ablation method, which requires specialized equipment.^81^ Metal oxide QDs, like SnO_2_ (ref. 85) and ZnSnO_3_/ZnO,^82^ use thermal decomposition or immersion methods, which are energy-intensive compared to PQDs' room-temperature synthesis. GQDs and NGQDs benefit from high surface area and tunable functionalization,^76,86^ but their synthesis (*e.g.*, hydrothermal methods^76^) is more complex than PQD's solution-based approaches. Chalcogenide QDs, such as CdS^74^ and PbS,^75^ require precise doping or coating, increasing fabrication complexity compared to PQD-metal oxide hybrids.^30,45^

**Fig. 12:**
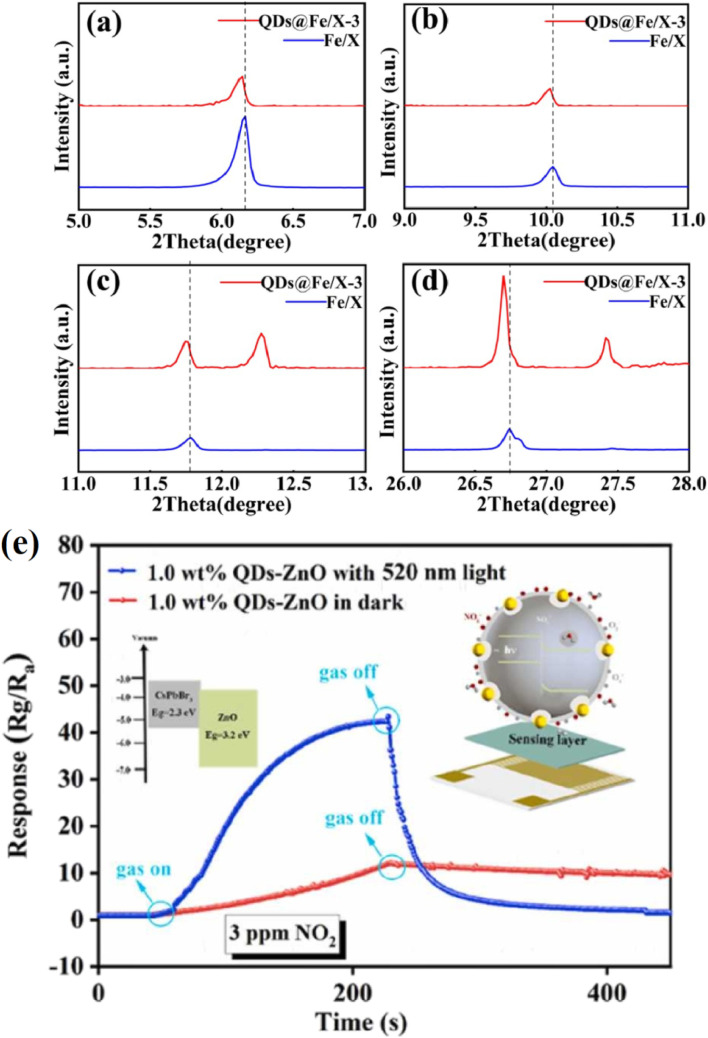
Locally magnified XRD patterns of the host and QDs@Fe/X-3 composites at selected 2*θ* regions (a–d), showing improved crystallinity and lattice ordering after PQD incorporation. Reprinted from ref. 25, with permission from RSC. (e) NO_2_ sensing performance of 1.0 wt% CsPbBr_3_ QDs–ZnO sensor under visible-light activation, demonstrating enhanced response and faster dynamics at room temperature. Reprinted from ref. 45, with permission from Elsevier, copyright 2022.

The locally amplified XRD patterns shown in Fig. 12a–d highlight the crystallographic improvements achieved after incorporating perovskite quantum dots (PQDs) into the zeolite-based framework.^25,45^ In all regions, the diffraction peaks of the QDs@Fe/X-3 composites are sharper and more intense compared to the pristine host, confirming enhanced lattice ordering and reduced microstrain. The low-angle reflections (5–7° and 9–11°) reveal the formation of well-organized host–guest structures, where PQDs are uniformly distributed within the zeolite matrix. The higher-angle regions (11–13° and 26–28°) correspond to characteristic perovskite crystal planes, whose narrowing and intensified peaks indicate that the embedded PQDs preserve their crystalline phase and experience fewer structural defects during encapsulation. This structural uniformity is key to achieving long-term optical and chemical stability under ambient conditions.^25^

The refined lattice order observed in the QDs@Fe/X-3 pattern reflects the strong interaction between the PQDs and the zeolite network, which effectively suppresses halide-vacancy formation and enhances electronic coupling at the interface. These improvements ensure that the PQDs retain high photoluminescence efficiency while remaining responsive to analyte molecules. When exposed to gases such as ammonia, the accessible surface sites on the stabilized PQDs allow rapid adsorption and desorption without compromising structural integrity. This reversible interaction enables a fast and repeatable change in PL intensity, demonstrating that the encapsulation strategy not only improves durability but also maintains the sensitivity required for reliable room-temperature gas detection.

Panel (e) illustrates the synergistic effect of PQD–metal oxide integration on the photoactivated sensing performance.^45^ The composite sensor composed of CsPbBr_3_ PQDs and ZnO microstructures exhibits a markedly enhanced response to trace NO_2_ concentrations under visible-light illumination compared with the dark condition. The improvement originates from the efficient charge transfer at the PQD–ZnO interface, where photoexcited carriers generated in the PQDs migrate to ZnO and participate in surface redox reactions. This process accelerates the activation of chemisorbed oxygen species, promoting faster adsorption and desorption of NO_2_ molecules. As a result, the sensor displays a high response amplitude and short response/recovery time at room temperature, emphasizing the benefit of visible-light-driven operation for low-power, high-sensitivity applications.

Overall, the XRD and photoresponse analyses presented in Fig. 12a–e demonstrate the dual advantages of PQD-based design strategies. Structural encapsulation within porous matrices improves intrinsic stability and preserves crystalline quality, while interfacial coupling with metal-oxide frameworks leverages the PQDs' strong light absorption and carrier mobility to amplify gas responses. These complementary design and fabrication approaches enable the development of robust, room-temperature sensors that combine long-term stability with enhanced sensitivity, aligning with the inherent advantages of PQD-based materials over conventional quantum-dot and metal-oxide systems.

### 6.4. Operational conditions and practicality

PQDs operate effectively at room temperature, a significant advantage over metal oxide QDs like ZnSnO_3_/ZnO (70 °C for HCHO^82^) or WO_3_ (elevated temperatures for CO^73^). TiO_2_ QDs^77^ and Ag@Cl-CQDs^78^ require UV or visible light, reducing portability compared to PQDs' passive operation.^25,45^ Chalcogenide QDs, like CdS@In_2_O_3_,^74^ achieve ppb-level NO_2_ detection but rely on MOF-derived structures, which are less scalable than PQD-zeolite systems.^25^

The comparative analysis of PQDs with other QD systems, such as metal oxides, chalcogenides, and carbon/graphene-based QDs, highlights their unique advantages and limitations in gas sensing applications. PQDs offer exceptional optoelectronic properties, including high PLQY (up to 90% for CsPbBr_3_) and tunable bandgaps (1.8–3.0 eV), enabling high sensitivity and selectivity for detecting toxic inorganic gases (*e.g.*, NO_2_, NH_3_, H_2_S), VOCs, oxygen, and humidity. However, challenges such as moisture sensitivity and lead toxicity necessitate innovative design strategies, which are compared against the robust stability of metal oxide QDs and the high surface area of carbon-based systems. Table 5 provides a detailed comparison of these systems, summarizing performance metrics (*e.g.*, sensitivity, LOD, response/recovery times), stability, operational conditions, and design features, offering insights into the competitive edge of PQDs for environmental, industrial, and biomedical gas sensing applications.

**Table 5:** Comparative analysis of PQDs and other QDs for gas sensing

Quantum dot system	Target gas	Response	LOD	Response/recovery time (s)	Selectivity/stability	Operating temperature	Design features	Ref.
PQDs: CsPbBr_3_ (filter paper)	NO	6 (*I*_N_2__/*I*_1000ppmNO_)	0.5 ppm	10/15	High *vs.* CO, CO_2_, NH_3_	Room temp. (25 °C)	Fluorescence-based, simple deposition	28
PQDs: CsPbBr_3_–ZnO	NO_2_	53 (@5 ppm)	100 ppb	63/40	High *vs.* CO, NH_3_, acetone	Room temp. (25 °C)	Metal oxide hybrid, heterojunction	45
PQDs: CsPbBr_3_-TBTO	H_2_S	0.58	250 ppb	278/730	High *vs.* NH_3_, SO_2_	Room temp. (25 °C)	Composite with TBTO, chemiresistive	24
PQDs: CH_3_NH_3_PbBr_3_ (silica aerogel)	SO_2_	2 (*I*_0_/*I*)	1 ppm	20/30	High *vs.* water vapor, CO_2_	Room temp. (25 °C)	Silica aerogel encapsulation, high surface area	63
PQDs: CsPbBr_3_ (zeolite X)	NH_3_	5 (*I*_0_/*I*)	0.5 ppm	15/20	98% PL retention (100 days)	Room temp. (25 °C)	Zeolite encapsulation, porous matrix	25
PQDs: CsPbBr_3_ (zinc acetylacetonate)	Ethanol	3 (*I*_0_/*I*)	3 ppm	8.5/13.9	High *vs.* water vapor	Room temp. (25 °C)	Ligand passivation, fluorescence-based	46
PQDs: CsPbBr_3_–In_2_O_3_	HCHO	31.4 (@2 ppm)	200 ppb	7/9	High *vs.* ethanol, NH_3_	Room temp. (25 °C)	Metal oxide hybrid, UV-activated	31
PQDs: CsPbBr_3_–In_2_O_3_	TEA	52.92 (@60 °C)	100 ppb	1/19	High *vs.* NH_3_, acetone	60 °C	Metal oxide hybrid, heterojunction	30
PQDs: Cs_2_AgBiBr_6_–Co_3_O_4_	Acetone	13 (@60 ppm)	500 ppb	7/27	High *vs.* ethanol, HCHO	Room temp. (25 °C)	Metal oxide hybrid, high conductivity	49
PQDs: FAPbI_3_ (rhodamine 110)	O_2_	12.7 (*R*_0_/*R*_100_)	0.05%	75/93	High *vs.* CO_2_, N_2_	Room temp. (25 °C)	Ratiometric with reference dye	29
PQDs: CsPbBr_3_–Pt(ii)	O_2_, NO	10.7 (O_2_), 2.7 (NO)	0.05% (O_2_), 0.5 ppm (NO)	10/15	High in mixed gases	Room temp. (25 °C)	Dual sensor, differential quenching	56
PQDs: EMT-CsPbBr_3_	Humidity	10 (*I*_0_/*I*)	0.1% RH	5/10	High *vs.* CO_2_, NH_3_	Room temp. (25 °C)	Fluorescence-based, high sensitivity	26
Metal oxide: WO_3_ (microwave-treated)	CO	5	4.6 ppm	30/50	High *vs.* oxidizing gases	Room temp. (25 °C)	Electrochemical synthesis, oxygen vacancies	73
Metal oxide: WO_3_ (microwave-treated)	NO_2_	10	1.5 ppm	25/55	High *vs.* other gases	Room temp. (25 °C)	Electrochemical synthesis, oxygen vacancies	73
Metal oxide: WO_3_ (microwave-treated)	NH_3_	8	0.35 ppm	28/45	High *vs.* other gases	Room temp. (25 °C)	Electrochemical synthesis, oxygen vacancies	73
Metal oxide: TiO_2_	H_2_S	25.12 (@500 ppb)	500 ppb	20/40	High *vs.* other gases	Room temp. (25 °C)	Microwave-assisted, surface defects	77
Metal oxide: ZnSnO_3_/ZnO	HCHO	613.3 (@100 ppm)	100 ppb	10/20	High *vs.* indoor pollutants	70 °C	Immersion method, heterojunction	82
Metal oxide: SnO_2_ (mesoporous)	H_2_S	30× increase	0.4 ppm	15/25	High *vs.* other gases	Room temp. (25 °C)	Thermal decomposition, mesoporous structure	85
Chalcogenide: CdS@In_2_O_3_	NO_2_	425.1 (@100 ppb)	10 ppb	10/20	High *vs.* other gases	Room temp. (25 °C)	MOF-derived, porous nanospheres	74
Chalcogenide: PbS@P3HT	NO_2_	50	200 ppb	10/20	High *vs.* other gases	Room temp. (25 °C)	Polymer doping, solution-processed	75
Chalcogenide: CdTe (HC-ARF)	NO_2_	20	∼0.1 ppm	5/10	High efficiency	Room temp. (25 °C)	Hollow-core fiber, fluorescence quenching	87
Chalcogenide: ZnSe: Cu@ZnS	Ethanol	10	1 ppm	10/15	Moderate (low w/o MWIR)	Room temp. (25 °C)	Microwave-assisted, core/shell structure	80
Carbon/GQDs: NGQD@SnO_2_	HCHO	100	0.01 ppb	5/10	High *vs.* other VOCs	Room temp. (25 °C)	Metal oxide hybrid, high sensitivity	86
Carbon/GQDs: NGQD@SnO_2_	NO_2_	150	0.1 ppb	5/10	High *vs.* other VOCs	Room temp. (25 °C)	Metal oxide hybrid, high sensitivity	86
Carbon/GQDs: NGQD@ZIF-8	Nitrobenzene	57% (@8–320 ppm)	0.44 ppm	10/15	High *vs.* other VOCs	Room temp. (25 °C)	Zeolitic framework, luminescence-based	83
Carbon/GQDs: Ag@Cl-CQDs	H_2_S	15	200 ppb	10/20	High (UV-activated)	Room temp. (25 °C)	Light-activated, Ag nanoparticle hybrid	78
Carbon/GQDs: MoS_2_/NGQD	NO_2_	82.4% (@ 50 ppm)	50 ppb	10/30	High *vs.* other gases	30 °C	Hydrothermal synthesis, 2D/0D heterostructure	76
Carbon/GQDs: CQD/Alq3	NO_2_	24% (@300 °C)	100 ppb	2/8	High *vs.* other gases	300 °C	Spin-coated, polymer composite	88

### 6.5. Comparison of PQD-based gas sensors with conventional sensor technologies

In order to contextualize the current progress PQD-based gas sensors, it is essential to compare their performance with conventional sensor systems, including metal oxide semiconductors (MOS), carbon-based nanomaterials, and polymeric sensors. Traditional MOS sensors such as ZnO, SnO_2_, and TiO_2_ have been widely employed due to their robustness and low cost; however, they typically require elevated operating temperatures (200–400 °C), which lead to high power consumption, slow recovery, and long-term drift.^14,15^ Moreover, their selectivity is generally poor because gas adsorption processes are governed by non-specific surface reactions. These limitations hinder their use in portable or flexible environmental monitoring devices.

Carbon-based materials such as graphene, carbon nanotubes (CNTs), and reduced graphene oxide (rGO) exhibit high electrical conductivity and mechanical flexibility, making them attractive for miniaturized sensors.^16,17^ Nevertheless, their sensing performance is often limited by weak gas adsorption and signal instability under humidity variations, requiring additional surface functionalization or doping strategies. Polymeric sensors, including polyaniline (PANI) and polypyrrole (PPy), are lightweight and flexible but typically suffer from irreversible swelling and aging effects under prolonged operation, which restrict their reproducibility and long-term reliability.^18–20^

In contrast, PQD-based sensors exhibit distinctive advantages derived from their quantum-confined structure and ionic lattice. PQDs such as CsPbBr_3_ and CH_3_NH_3_PbI_3_ demonstrate high photoluminescence quantum yield (PLQY, up to 90%), narrow emission linewidths (12–40 nm), and high carrier mobility (up to 4500 cm^2^ V^−1^ s^−1^), enabling both fluorescence- and chemiresistive-type sensing with remarkable sensitivity and selectivity.^24,30,33^ Unlike MOS or CNT sensors, PQDs can operate efficiently at room temperature, which significantly reduces power consumption and allows their integration into flexible and wearable electronics. Their tunable bandgap and surface chemistry facilitate targeted interactions with oxidizing (NO_2_, O_2_) or reducing (NH_3_, H_2_S) gases, offering detection limits in the sub-ppm or even ppb range—often surpassing conventional systems.^25,31,35^

Furthermore, the solution-processable nature of PQDs enables low-cost fabrication using spin-coating, drop-casting, or inkjet printing, which contrasts with the high-temperature sintering required for MOS materials. Recent studies have demonstrated stable PQD-based sensors embedded in silica aerogels or zeolites with prolonged operational lifetimes exceeding 100 days, addressing the durability challenges that typically affect polymeric or carbon-based devices.^25,27,63^ Overall, PQD-based gas sensors represent a new generation of nanostructured sensing platforms combining ambient-condition operation, superior selectivity, high signal reproducibility, and facile processability, establishing them as strong candidates for next-generation environmental monitoring and smart sensing technologies.^18,63^

## 7. Challenges and advancements in perovskite QD-based gas sensing

PQDs offer exceptional PLQY, tunable bandgaps, and high surface-to-volume ratios for sensing toxic inorganic gases, VOCs, oxygen, and humidity. Despite their promise, challenges like environmental instability, limited selectivity, lead toxicity, and fabrication complexity hinder practical deployment. This section analyzes these limitations, proposes advanced strategies to address them, and outlines future directions to enhance PQD-based gas sensors, avoiding mechanistic details (Section 3) and performance metrics (Section 4).

### 7.1. Primary limitations of PQD-based gas sensors

#### 7.1.1. Environmental instability

The ionic ABX_3_ perovskite lattice makes PQDs vulnerable to moisture, heat, and light. Moisture coordinates with surface ions, causing structural dissociation and significant PL loss in humid conditions (*e.g.*, 80% RH).^32,89^ Thermal instability, due to low formation energies (∼0.5 eV for CsPbBr_3_), leads to decomposition above 100 °C, limiting use in high-temperature industrial settings.^32^ Photo-induced degradation under UV or visible light creates halide vacancies, reducing PLQY, particularly in fluorescence-based sensors.^90–92^ Reactive analytes like H_2_S can irreversibly disrupt the lattice, forming non-luminescent compounds.^54^

#### 7.1.2. Limited selectivity in complex gas mixtures

PQDs often exhibit non-specific interactions at surface defects, reducing selectivity in multi-gas environments. For instance, CsPbBr_3_ PQDs show strong fluorescence response to NH_3_ but are sensitive to water vapor, affecting accuracy in humid conditions.^26^ Chemiresistive hybrids like CsPbBr_3_–ZnO require surface engineering to distinguish NO_2_ from CO or NH_3_.^45,93^ In biomedical applications, such as acetone detection for diabetes, interference from ethanol or humidity complicates results,^46^ limiting reliability in complex settings.

#### 7.1.3. Toxicity of lead-based PQDs

Lead-based PQDs, such as CsPbBr_3_, raise environmental and health concerns due to potential leakage during degradation, particularly in large-scale or biomedical applications.^32,94^ Lead-free alternatives like Cs_2_AgBiBr_6_ have lower PLQY (∼50% *vs.* 90% for CsPbBr_3_) and reduced stability, compromising sensing performance.^49^ Balancing toxicity reduction with optoelectronic efficiency remains a challenge for sustainable deployment.

#### 7.1.4. Fabrication complexity and scalability

Integrating PQDs into stable sensors often involves complex post-synthesis treatments, such as encapsulation in zeolites or ligand exchange, increasing costs.^25,95^ Hybrid systems with metal oxides require multi-step fabrication, hindering scalability compared to simpler systems like carbon-based QDs.^45,86^ These complexities challenge commercial viability, particularly for large-scale environmental monitoring.

### 7.2. Advanced strategies for overcoming limitations

#### 7.2.1. Novel encapsulation for enhanced stability

Advanced encapsulation enhances PQD durability. Fe-doped zeolite X protects CsPbBr_3_ PQDs, enabling stable NH_3_ sensing in humid conditions by shielding against moisture while allowing gas diffusion.^25,97^ Silica aerogels stabilize CH_3_NH_3_PbBr_3_ PQDs for SO_2_ detection, leveraging hydrophobicity to prevent degradation.^63^ Polymer-based encapsulation, such as CsPbBr_3_ in EVA, supports methanol sensing and offers flexibility for wearable devices.^27^ Future strategies could explore metal–organic frameworks (MOFs) with tunable porosity to optimize stability and gas access.

#### 7.2.2. Surface engineering and machine learning for selectivity

Surface engineering with tailored ligands, like zinc acetylacetonate, reduces water vapor interference for ethanol detection, with machine learning analyzing PL patterns to enhance specificity.^46^ Gas-specific receptors, such as thiol-based ligands for H_2_S, can minimize cross-sensitivity. Hybrid systems with 2D materials like MoS_2_, inspired by MoS_2_/NGQD hybrids, suggest potential for high selectivity through increased surface area.^76^ Machine learning can distinguish gas signatures in complex mixtures, critical for breath analysis or industrial monitoring.^96^

#### 7.2.3. Lead-free PQD development

Lead-free PQDs, such as Cs_2_AgBiBr_6_, require optimization to match lead-based performance. Ion doping with Mn^2+^ or Bi^3+^ enhances lattice stability, as seen in acetone-sensing Cs_2_AgBiBr_6_.^49^ Microwave-assisted synthesis improves crystallinity, boosting PLQY in lead-free systems.^32^ Exploring double-perovskite or hybrid organic-inorganic structures could yield eco-friendly, high-performance sensors.

#### 7.2.4. Streamlined fabrication for scalability

Room-temperature co-precipitation produces uniform PQDs with high PLQY, reducing complex post-synthesis steps.^32^ Automated ligand exchange in flow-based systems streamlines passivation. Flexible substrates, like polymers used in CH_3_NH_3_PbBr_3_ PQDs for NH_3_ sensing, support scalable production of portable sensors.^55^ Standardized protocols can ensure cost-effective industrial adoption.

### 7.3. Future perspectives for PQD-based gas sensing technologies

#### 7.3.1. Integration with emerging technologies

Integrating PQDs with IoT enables real-time environmental monitoring across large areas, leveraging low-power operation. Flexible substrates, as in CH_3_NH_3_PbBr_3_ PQDs, support wearable sensors for industrial or biomedical use.^55,98^ Machine learning, applied to ethanol detection, could enable rapid gas identification in complex environments.^46^

#### 7.3.2. Multifunctional and self-powered sensors

Multifunctional sensors detecting multiple gases, like CsPbBr_3_–Pt(ii) for O_2_ and NO, show promise for multi-analyte detection.^56^ Ratiometric designs with reference emitters, such as FAPbI_3_ for O_2_, should expand to other gases.^78^ Self-powered sensors, integrating PQDs with photovoltaic materials, could enable energy-independent devices for remote monitoring.

#### 7.3.3. Advanced heterostructures

Novel heterostructures with 2D materials like graphene or MXenes could enhance conductivity and stability, building on MoS_2_/NGQD systems.^76^ These materials offer high surface areas, potentially surpassing metal oxide hybrids like CsPbBr_3_–In_2_O_3_.^30^

#### 7.3.4. Regulatory compliance and sustainability

Prioritizing lead-free PQDs and safe disposal protocols addresses toxicity concerns. Life-cycle assessments can guide eco-friendly designs, ensuring compliance with environmental regulations. Table 6 outlines challenges and strategies for PQD-based gas sensors.

**Table 6:** Challenges and strategies for PQD-based gas sensors

Challenge	Description	Strategy	Future direction	Ref.
Environmental instability	Susceptibility to moisture, heat, light	Zeolite/silica encapsulation, polymer coatings	MOFs with tunable porosity	25 and 63
Limited selectivity	Non-specific interactions in multi-gas settings	Tailored ligands, machine learning, 2D material hybrids	Gas-specific receptors, neuromorphic computing	46 and 76
Lead toxicity	Environmental/health risks from Pb-based PQDs	Lead-free PQDs, ion doping	Double-perovskites, hybrid compositions	49
Fabrication complexity	Complex post-synthesis treatments	Room-temperature synthesis, automated ligand exchange	Scalable substrates, standardized protocols	32 and 55
Slow response dynamics	Longer response/recovery times for some analytes (*e.g.*, H_2_S)	Optimized surface passivation, nanostructured matrices	High-surface-area heterostructures	24
Integration complexity	Challenges in combining PQDs with IoT or wearable platforms	Flexible substrates, low-power designs	IoT-enabled sensor arrays, neuromorphic systems	55 and 96
Energy consumption	High power needs for continuous operation	Self-powered systems with photovoltaic integration	Piezoelectric or solar-driven sensors	97
Recycling challenges	Environmental impact of PQD disposal	Eco-friendly designs, recycling protocols	Life-cycle assessments, sustainable materials	32

## 8. Conclusion

PQDs have emerged as exceptional next-generation materials for gas sensing applications, owing to their unique optoelectronic tunability, structural flexibility, and high surface reactivity. This review has comprehensively analyzed the synthesis strategies, fundamental mechanisms, characterization techniques, and performance trends that define PQD-based gas sensors. Through this integrated approach, it provides a unified understanding of how synthesis methodology and surface chemistry determine the sensing behavior and operational stability of PQDs. The novelty of this review lies in its holistic correlation between synthesis, structure, and sensing functionality, presenting a comparative evaluation of PQD-based sensors with conventional counterparts such as metal-oxide, carbon-based, and polymeric systems. This comparative insight highlights the superior sensitivity, selectivity, and room-temperature performance of PQD-based platforms, while also emphasizing the remaining scientific challenges that limit their commercialization. Moreover, by combining advances in photoluminescence-based and chemiresistive sensing, this review bridges the gap between material chemistry and device engineering—an approach not systematically addressed in earlier literature.

In terms of scope and contribution, this review not only consolidates the current state of PQD-based gas-sensing research but also defines a forward-looking roadmap for the field. It identifies unresolved challenges such as intrinsic instability in humid and oxidative environments, lead toxicity, and scalability limitations, while summarizing emerging strategies for mitigation. These include ligand engineering, heterostructure formation, ion doping, and encapsulation within robust inorganic or polymer matrices. Furthermore, the review extends its perspective to new research directions, including the design of eco-friendly lead-free PQDs, machine learning-assisted signal processing, and integration of PQDs with wearable and flexible sensing systems. Overall, PQDs offer a transformative platform for gas sensing, capable of outperforming many existing nanomaterial-based technologies in terms of detection sensitivity, operating temperature, and device adaptability. The comprehensive framework presented here—linking synthesis control, optoelectronic behavior, and sensing performance—provides valuable insights for researchers seeking to optimize PQD-based devices for environmental, industrial, and biomedical applications. Continued interdisciplinary research across chemistry, materials science, and engineering will be essential to translate the remarkable laboratory achievements of PQD-based sensors into robust, real-world technologies.

## Author contributions

Suleiman Ibrahim Mohammad and Ahmad Mohebi conceptualized the review, supervised the project, and prepared the original draft; A. K. Kareem, Fadhil Faez Sead, and D. S. Jayalakshmi conducted literature investigation and data curation; Zyad Shaaban and Sanjeev Kumar performed formal analysis and validation; M. Sudhakara Reddy and Satish Choudhury provided resources and visualization; Asokan Vasudevan managed project administration; all authors contributed to writing, review, and editing of the manuscript and have approved the final version.

## Conflicts of interest

The authors declare no conflict of interest.

## Data Availability

No primary research results, software or code have been included and no new data were generated or analysed as part of this review.
